# Impact of pathological factors on survival in patients with upper tract urothelial carcinoma: a systematic review and meta-analysis

**DOI:** 10.1590/S1677-5538.IBJU.2020.1032

**Published:** 2021-04-20

**Authors:** Gopal Sharma, Anuj Kumar Yadav, Tarun Pareek, Pawan Kaundal, Shantanu Tyagi, Sudheer Kumar Devana, Shrawan Kumar Singh

**Affiliations:** 1 Postgraduate Institute of Medical Education and Research Department of Urology Chandigarh India Department of Urology, Postgraduate Institute of Medical Education and Research, Chandigarh, India

**Keywords:** Carcinoma, Transitional Cell, Pathology, Prognosis

## Abstract

**Introduction:**

There is an ongoing need to identify various pathological factors that can predict various survival parameters in patients with upper tract urothelial carcinoma (UTUC). With this review, we aim to scrutinize the impact of several pathological factors on recurrence free survival (RFS), cancer-specific survival (CSS) and overall survival (OS) in patients with UTUC.

**Materials and Methods:**

Systematic electronic literature search of various databases was conducted for this review. Studies providing multivariate hazard ratios (HR) for various pathological factors such as tumor margin, necrosis, stage, grade, location, architecture, lymph node status, lymphovascular invasion (LVI), carcinoma in situ (CIS), multifocality and variant histology as predictor of survival parameters were included and pooled analysis of HR was performed.

**Results:**

In this review, 63 studies with 35.714 patients were included. For RFS, all except tumor location (HR 0.94, p=0.60) and necrosis (HR 1.00, p=0.98) were associated with worst survival. All the pathological variables except tumor location (HR 0.95, p=0.66) were associated with worst CSS. For OS, only presence of CIS (HR 1.03, p=0.73) and tumor location (HR 1.05, p=0.74) were not predictor of survival.

**Conclusions:**

We noted tumor grade, stage, presence of LVI, lymph node metastasis, hydronephrosis, variant histology, sessile architecture, margin positivity and multifocality were associated with poor RFS, CSS and OS. Presence of CIS was associated with poor RFS and CSS but not OS. Tumor necrosis was associated with worst CSS and OS but not RFS. Tumor location was not a predictor of any of the survival parameters.

## INTRODUCTION

Upper tract urothelial carcinomas (UTUCs) are rare but aggressive malignancies, accounting for about 5-10% of all urothelial cancers (1). They have an estimated incidence of around 2 cases per 100.000 person-year in the United States (1, 2). Radical nephroureterectomy with bladder cuff excision with or without lymph node dissection is the cornerstone for the management of these cases (3). Until recently, data on the use of systemic chemotherapy either in the adjuvant or neoadjuvant setting was based on small retrospective studies (4). Only in a recently reported phase III randomized controlled trial (RCT), definite survival advantage with adjuvant chemotherapy has been shown (5). Multiple prognostic factors have been implicated with survival outcomes in patients with UTUCs. These prognostic factors have been conveniently divided into clinical, surgical and pathological factors (3, 6). Besides, several molecular markers have been associated with prognosis in UTUCs in various single or multicenter studies (6, 7). The purpose of these prognostic markers is to identify patients with aggressive disease and institute prompt adjuvant therapy.

Some of the pathological factors such as tumor stage, lymph node metastasis, tumor grade, lymphovascular invasion (LVI) have been consistently reported as predictors of all the survival outcomes i.e. recurrence-free survival (RFS), cancer-specific survival (CSS) and overall survival (OS) (6). The literature on the other pathological factors such as the presence of tumor necrosis (8, 9), carcinoma in situ (CIS) (10-12), variant histology (13-19) and multifocality (20-22) as prognostic factors for survival in UTUC is still conflicting concerning for different survival outcomes. Data for these pathological factors have been mostly derived from retrospective observational studies. Some of these pathological variables have been individually evaluated in systematic reviews as a predictor of survival parameters (23-25). However, these studies had multiple limitations (including data from overlapping patient population studies, limited search) and were not methodologically adequate (24, 25). Furthermore, there has been only one review that assessed various clinical-pathological factors associated with intravesical recurrence in patients with UTUC (26). To the best of our knowledge, there hasn’t been a systematic review examining all the pathological variables for all the clinically essential survival outcomes i.e. CSS, RFS and OS following surgical management for patients with UTUC. Thus, this systematic review aimed to scrutinize the survival predictability of various pathological variables (such as tumor necrosis) for which literature is still conflicting and generate pooled hazard ratios (HR) for other pathological factors for all the relevant survival parameters (OS, CSS and RFS) in a single study.

## MATERIALS AND METHODS

### Study Design

With this study, we comprehensively explored all the available literature regarding various pathological factors implicated in the survival of patients with UTUCs. We included all the studies where data on multivariable analysis predicting various survival outcomes such as CSS, OS and RFS were available. From each of these studies, HR for different pathological variables was extracted for quantitative analysis. While conducting this review standard preferred reporting items for systematic reviews and meta-analysis (PRISMA) guidelines (27) were followed. The study protocol was registered with PROSPERO (CRD42020184885).

### Search Strategy and selection criteria

The literature search for this review was conducted by two review authors independently (GS & TP). Multiple electronic databases such as Pubmed/Medline, Scopus, Embase, CENTRAL and Web of Science were used for conducting the literature search. The literature search was conducted from the date of inception of these databases till the last search on 29th March 2020. Following filters were applied [Species-Humans] and [Language-English]. Additional articles were sought from the articles selected for the full-text review.

We followed the PICO (patient/population, intervention, control, outcome) methodology to design our search strategy.

Patient/population: Upper tract urothelial carcinoma, upper tract urothelial cancer, UTUC

Control/Intervention: stage, grade, lymphovascular invasion, LVI, tumor necrosis, margin, tumor margin, carcinoma in situ, CIS, multifocality, architecture, sessile, pathology, pathological, variant histology, tumor location.

Outcome: prognosis, prognostic, survival.

Both key words and meshed terms were used to develop the search strategy. Key words used for this study were “upper tract urothelial carcinoma” OR “upper tract urothelial cancer” OR “UTUC” AND “stage” OR “grade” OR “lymphovascular invasion” OR “LVI OR “tumor necrosis” OR “margin” OR “tumor margin” OR “carcinoma in situ” OR “CIS” OR “multifocality” OR “architecture” OR “sessile” OR “pathology” OR “pathological” OR “variant histology” OR “location” AND “prognosis” OR “prognostic” OR “survival” OR “outcome”.

**Figure 1:**
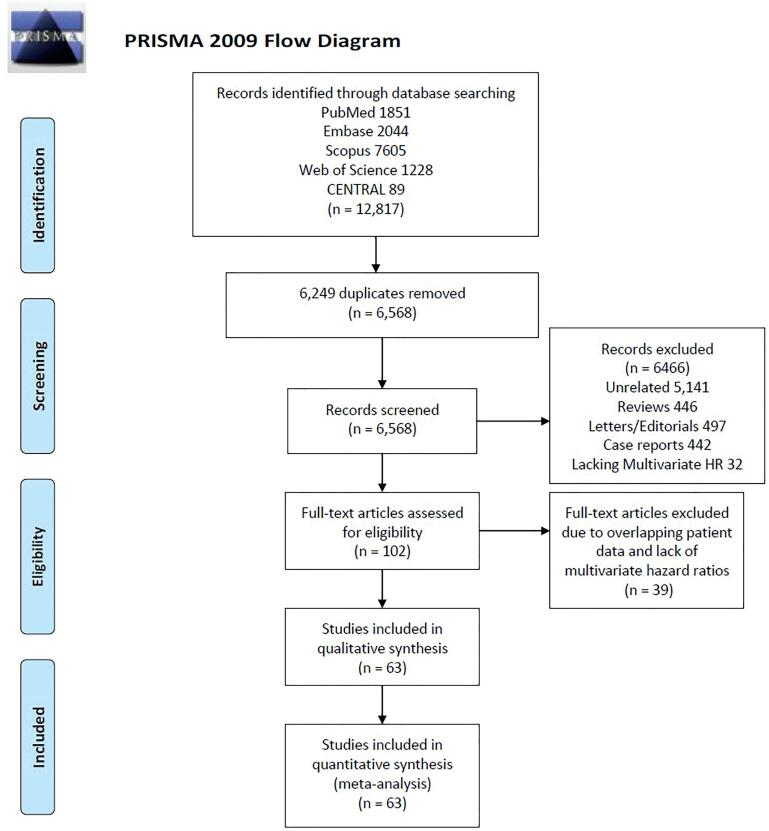
PRISMA flow-chart depicting search strategy used for conducting this review.

The search strategy used for PubMed has been provided in supplementary file S1 (Appendix-1).

### Statistical Analysis

Forest plots were used to perform quantitative analysis of multivariate HR and generate pooled HR to describe relation between a particular pathological variable and survival parameters (CSS, OS and RFS). For T- stage of the tumor we performed a pooled analysis of HR of those studies that only compared stage T_3_ and T_4_ stages against T_is_, T_1_ and T_2_. For assessment of grade, we used HR describing the relation between high grade and low-grade tumor for survival outcomes. Similarly, pooled HRs was generated for variant histology (absence or presence), tumor necrosis (absence or presence), LVI (absence or presence), multifocality (absence or presence), CIS (absence or presence), margin status (negative or positive), tumor architecture (papillary or sessile), tumor location (ureter vs. renal pelvis), and lymph node metastasis (absence or presence) in relation to various survival parameters (CSS, OS and RFS). Statistical analysis was performed using the Cochrane Collaboration review manager software RevMan 5.2™ (the Cochrane Collaboration, Copenhagen, Denmark). Chi^2^ and I^2^ tests were used to assess heterogeneity across each variable in the quantitative analysis. A p-value <0.10 was used to indicate significant heterogeneity and in such a case Random effect model was used. Whereas, p-value was >0.10 signifies absence of statistical heterogeneity and in such a case fixed-effects model (Mantel-Haenszel method) was used. A p-value of <0.05 was considered statistically significant.

### Outcomes

Survival parameters (CSS, OS & RFS) were assessed according to various pathological factors such as stage (T_is_, T_A_, T_1_ & T_2_ vs. T_3_ & T_4_), tumor grade (low versus high), variant histology (absence vs. presence), tumor necrosis (absence vs. presence), LVI (absence vs. presence), multifocality (absence vs. presence), tumor location (ureter vs. renal pelvis), CIS (absence vs. presence) and margin status (negative vs. positive), tumor architecture (papillary vs. sessile) and lymph node metastasis (absence vs. presence). Recurrence-free survival was defined as the absence of extraluminal metastasis (local surgical site recurrence, distant metastasis, local and distant metastatic lymph nodes). Studies including only bladder or contralateral upper urinary tract were not included in recurrences free survival calculations. We initially also planned to study tumor size variable, however pooled analysis was not possible due to lack of consistent data for this parameter. Some studies had reported tumor size as a continuous variable and others as a categorical variable with variable cut-offs. Impact of other clinical parameters such as mode of surgery (open or minimally invasive) or chemotherapy (adjuvant and neoadjuvant) were not a part of this study.

### Quality assessment

We used the Newcastle-Ottawa quality assessment scale (NOS) for the quality assessment of the studies included in this review. Using this scale quality assessment of non-randomized studies was done based upon selection and comparability of study groups and ascertainment of the primary outcome in the two groups. A study can be awarded a maximum of 9 stars, studies with >5 stars are considered to be of good quality. Quality assessment was performed by two review authors (GS & TP) independently and the help of other authors was sought in case of discrepancy of results (AKR & PMK).

## RESULTS

### Search strategy and study selection

Using various electronic databases mentioned above, a total of 12.817 articles were extracted of which 6.249 duplicate citations were removed. A total of 6.568 articles underwent initial title and abstract screening of which 6.466 articles were excluded for not meeting the inclusion criteria. Full-text reviews of 102 articles were performed of which 39 articles were removed due to overlapping patient data and lack of multivariate HR. For the final analysis, 63 studies were included in this meta-analysis (supplementary file S2 – Appendix-1).

### Study characteristics and quality assessment

A total of 63 studies were included in the final analysis with 35.714 patients. All the included studies were retrospective in nature and 30 were multicenter. The duration of follow-up and variables adjusted in multivariate analysis were variable in all the studies (Supplementary Table-2). Further details on age, stage, LVI, tumor necrosis, factors controlled in multivariate analysis and survival parameters studies across the studies have been provided in supplementary Table- S3 (Appendix-1). Quality assessment as performed using NOS revealed stars ranging from 6-8, with 26, 34 and 3 studies being awarded 6, 7 and 8 stars respectively.

### Pooled analysis

Tumor location (Ureter versus renal pelvis)

Multivariate HRs for tumor location concerning to RFS, CSS and OS were available from 3, 5 and 3 studies respectively. Pooled HR for the RFS, CSS and OS were 0.94 (0.75, 1.18), 0.95 (0.78, 1.17) and 1.05 (0.80, 1.36) respectively. There was no statistically significant difference for the pooled HR for any of the survival outcomes.

### Stage of the tumor

Of all the studies, data comparing T3 and T4 to lower stages of the tumor was available from 14, 22 and 16 studies for RFS, CSS and OS respectively. Higher tumor stage was significant predictor of recurrence (HR 2.43, 95% CI (1.86, 3.17), p <0.00001), poor CSS (HR 2.69, 95% CI (2.28, 3.18), p <0.00001) and poor OS (HR 2.45, 95% CI (2.19, 2.73), p <0.00001).

### Grade of the tumor

Data on comparison for the high-grade to the low-grade tumor was available for RFS, CSS and OS from 22, 38 and 23 studies respectively. Higher tumor grade was associated with poor survival outcomes with significantly higher HRs i.e. RFS (HR 1.39, 95% CI (1.17, 1.65), p <0.00001), CSS (HR 1.69, 95% CI (1.45, 1.98), p <0.00001) and OS (HR 1.60, 95% CI (1.44, 1.77), p <0.00001) (Appendix-2).

### LVI and positive lymph nodes

The presence or absence of LVI for RFS, CSS and OS were noted in 27, 36 and 21 studies respectively, whereas data on the positivity of lymph nodes was available from 23, 36 and 21 studies for RFS, CSS and OS respectively. Both presence of LVI and lymph node positivity were associated with significantly higher HRs for all three survival parameters. Pooled HRs for LVI and positive lymph nodes were 1.73 (95% CI (1.47, 2.03) and 2.22 (95% CI (1.88, 2.62) respectively for RFS. Pooled HRs for CSS was 2.03 (95% CI (1.74, 2.36) and 2.24 (95% CI (1.99, 2.52) for LVI and lymph node positivity. For OS pooled HRs were 1.60 (95% CI (1.37, 1.87) for LVI and 2.02 (95% CI (1.72, 2.39) for positive lymph nodes (Appendix-2).

### Architecture of the tumor (papillary versus sessile)

Quantitative data on multivariate HR for tumor architecture was available from 12, 12 and 8 studies for RFS, CSS and OS respectively. Sessile tumor architecture was associated with significantly higher HR for RFS (1.48 (95% CI (1.20, 1.83)), CSS (1.47 (95% CI (1.22, 1.76)) and OS (1.58 (95% CI (1.26, 1.99)) (Appendix-2).

### Multifocality and presence of CIS

The presence of multiple tumors and CIS were associated with significantly higher HR for all the survival parameters except for one (CIS for OS). For RFS pooled HR was 1.14 (95% CI (1.02, 1.29) for CIS and 1.52 (95% CI (1.13, 2.04) for multifocality, for CSS pooled HR were 1.21 (95% CI (1.06, 1.38) for CIS and 1.33 (95% CI (1.12, 1.59) for multifocality, for OS pooled HR were 1.05 (95% CI (0.87, 1.25) for CIS and 1.50 (95% CI (1.28, 1.76) for multifocality (Appendix-2).

### Tumor margin positivity and necrosis

From the pooled analysis of all the studies with available data on surgical margin status, we noted positive surgical margin was associated with the worst RFS (HR 1.38, 95%CI (1.20, 1.59), p <0.00001), CSS (HR 1.59, 95% CI (1.36, 1.87), p <0.00001) and OS (HR 1.71, 95% CI (1.34, 2.19), p <0.0001). Presence of tumor necrosis was significant predictor of poor CSS (HR 1.47, 95% CI (1.08, 1.99), p=0.01) and OS (HR 1.77, 95% CI (1.05, 2.95), p=0.03) but not RFS (HR 1.00, 95% CI (0.86, 1.16), p=0.98).

### Variant histology

As previously mentioned, some studies have described specifically the subtype of variant histology whereas others have not. The presence of variant histology was associated with significantly worst survival parameters i.e. RFS (HR 1.48, 95% CI (1.31, 1.66), p <0.00001), CSS (HR 1.86, 95% CI (1.51, 2.30), p <0.00001) and OS (HR 1.74, 95% CI (1.47-2.05), p <0.00001) (Appendix-2).

## DISCUSSION

UTUCs are considered to be one of the most aggressive urological malignancies, around 60% of cases have muscle invasion compared to 15-25% of the bladder tumors at diagnosis (28, 29). One of the vexing issues associated with their management is the high rates of the bladder (22-47%) and contralateral upper tract (2-6%) recurrences following treatment (30-32). To prognosticate and intensify the treatment regimens according to the patient-specific risk factors, a risk-adapted classification has been provided in the European Association of Urology (EAU) guidelines (3). Many pathological factors are considered important prognostic factors and guidelines recommend explicit reporting of such elements in the final pathology. As previously noted, the role of some of the pathological factors as an independent predictor is not clear as the data are conflicting. In a previous meta-analysis by Seisen et al. (26), assessing risk for intravesical recurrence for various clinic-pathological factors; the authors noted ureter tumor location, multifocality, pathological T stage, tumor necrosis and positive surgical margin were independent predictors of intravesical recurrence and, LVI, concomitant CIS, tumor grade, and positive lymph node status were not identified as independent predictors of intravesical recurrence. The above mentioned-review despite being exhaustive and methodologically sound was limited by the fact that they only studied the risk factors for intravesical recurrence. Thus, the clinical relevance of this review becomes more as no previously conducted review has examined all the pathological factors at the same time for all the survival outcomes.

In this large systematic review, a total of 63 studies with 35.714 patients were included. Most of the studies included in this review were multicenter and retrospective case series. Quality assessment performed using NOS and all the studies scored more than 6 on this scale implying that all the studies were of adequate quality. However, caution should be exerted while interpreting the results of this review as the results have been pooled from retrospective case series which are inherently at risk of bias. With the paucity of properly conducted prospective studies, this study remains the best evidence available so far in the literature.

In this study, pooled analysis for survival outcomes (RFS, CSS and OS) for 11 pathological variables was performed (Table-1). For RFS, all the pathological variables except tumor location and necrosis were associated with significantly higher pooled HRs. Thus, for RFS tumor location and necrosis were not predictors of survival. For CSS, all the variables except tumor location were identified as independent predictors and for OS all but tumor location and presence of CIS were independent predictors. In a previous meta-analysis by Ku et al. (33), authors noted LVI to be a predictor of RFS and CSS but not OS, on the contrary, we noted LVI to be a predictor of all the survival parameters (CSS, OS, RFS). Compared to the study by Ku et al. (33) our study is much larger and most updated. In another meta-analysis, Fan et al. (24) noted sessile tumor architecture to be associated with worst the RFS and CSS, however, authors did not include OS in the analysis. Regarding presence of CIS, our findings are similar to a previous meta-analysis by Gao et al. (25), who also noted CIS to be associated with poor RFS and CSS but not OS. These two previously mentioned meta-analysis by Fan et al. (24) and Gao et al. (25) were of limited methodological quality as they contained studies with overlapping patient populations. For the presence of variant histology (23), our findings are similar to a previously reported meta-analysis on the topic by Mori et al. Another important point noted in our study is that tumor location is not an independent predictor of survival which is contrary to few individual studies (34, 35) in which ureter location was identified as an independent predictor of poor survival outcomes. However, we acknowledge that the pooled analysis for the location was derived from a handful number of studies which can be its limitation. Literature regarding tumor necrosis as an independent prognostic factor is controversial (8, 9). From our pooled analysis, we noted tumor necrosis to be associated with the worst CSS and OS but not RFS. Even after an exhaustive literature search, we could not find any systematic review reporting data on grade, stage, lymph node status, tumor location, tumor necrosis and margin status as predictors of survival in patients with UTUCs. Thus, our study is the first systematic review to provide pooled analysis for the above-mentioned pathological variables.

**Table 1:** Survival analysis for various pathological factors with their pooled analysis.

Recurrence free survival								
S.no.	Variable	Number of studies	Chi^2^	I^2^	Model	Pooled HR	95% CI	p-value
1	Tumor location (ureter vs. pelvic)	3	2.99	33%	IV Fixed	0.94	0.75,1.18	0.60
2	T stage	14	60.11	78%	Random	2.43	1.86-3.17	<0.00001
3	Grade	22	46.86	55%	IV, Random	1.39	1.17, 1.65	0.0002
4	LVI	27	121.1	79%	IV, Random	1.73	1.47, 2.03	<0.00001
5	LN positivity	23	62.29	65%	IV, Random	2.22	1.88, 2.62	<0.00001
6	Architecture	12	43.27	75%	IV, Random	1.48	1.20, 1.83	0.0002
7	CIS	9	6.24	0%	IV Fixed	1.14	1.02, 1.29	0.02
8	Multifocality	7	22.39	73%	IV, Random	1.52	1.13, 2.04	0.006
9	Margin	9	7.93	0%	IV Fixed	1.38	1.20, 1.59	<0.00001
10	Necrosis	4	5.35	44%	IV, Random	1.00	0.86, 1.16	0.98
11	Variant Histology	11	16.27	26%	Fixed	1.48	1.31-1.66	<0.00001

Cancer specific survival								
S.no.	Variable	Number of studies	Chi^2^	I^2^	Model	Pooled HR	95% CI	p-value
1	Tumor location (ureter vs. pelvic)	5	3.66	0%	IV, Fixed	0.95	0.78,1.17	0.66
2	T stage	22	34.07	38%	Random	2.69	2.28-3.18	<0.00001
3	Grade	38	81.55	55%	IV, Random	1.69	1.45, 1.98	<0.00001
4	LVI	36	117.1	70%	IV, Random	2.03	1.74, 2.36	<0.00001
5	LN positivity	36	52.69	35%	IV, Random	2.24	1.99, 2.52	<0.00001
6	Architecture	12	22.9	52%	IV, Random	1.47	1.22, 1.76	<0.0001
7	CIS	17	14.31	0%	IV, Fixed	1.21	1.06, 1.38	0.004
8	Multifocality	14	27.7	53%	IV, Random	1.33	1.12, 1.59	0.001
9	Margin	12	13.53	19%	IV, Fixed	1.59	1.36, 1.87	<0.00001
10	Necrosis	8	20.14	65%	IV, Random	1.47	1.08, 1.99	0.01
11	Variant Histology	20	60.66	64%	IV, Random	1.86	1.51-2.30	<0.00001

Overall survival								
S.no.	Variable	Number of studies	Chi^2^	I^2^	Model	Pooled HR	95% CI	p-value
1	Tumor location (ureter vs. pelvic)	3	2.63	24%	IV, Fixed	1.05	0.80,1.36	0.74
2	T stage	16	10.86	0%	IV, Fixed	2.45	2.19-2.73	<0.00001
3	Grade	23	14.28	0%	IV, Fixed	1.60	1.44, 1.77	<0.00001
4	LVI	21	60.48	67%	IV, Random	1.60	1.37, 1.87	<0.00001
5	LN positivity	21	38.46	48%	IV, Random	2.02	1.72, 2.39	<0.00001
6	Architecture	8	19.73	65%	IV, Random	1.58	1.26, 1.99	<0.0001
7	CIS	8	2.8	0%	IV, Fixed	1.05	0.87, 1.25	0.63
8	Multifocality	10	8.75	0%	IV, Fixed	1.50	1.28, 1.76	<0.00001
9	Margin	10	21.07	57%	IV, Random	1.71	1.34, 2.19	<0.0001
10	Necrosis	5	8.5	53%	IV, Random	1.77	1.05, 2.95	0.03
11	Variant Histology	13	21.01	43%	IV, Random	1.74	1.47-2.05	<0.00001

**HR=**Hazard ratio; **CIS**= carcinoma in situ, **LN** = lymph node; **LVI**= lymphovascular invasion; **IV**= Inverse variance

## LIMITATIONS

There are multiple limitations of this study that needs to be highlighted. We acknowledge that the studies included in this study were observational studies that have inherent selection bias. Furthermore, the likelihood of reporting bias cannot be completely ruled out as negative trials have lower chances of publication. We also noted significant heterogeneity in the analysis of some pathological factors for survival parameters. For accounting for heterogeneity in the model we used the random-effects model. Since our review focused only on the impact of various pathological factors on oncological outcomes, we were not able to control for other multiple confounding factors. Firstly, different types of surgical methods have been employed for the treatment (open or laparoscopic or segmental ureterectomy). Secondly, lymph node dissection was performed in some and not in others. Thirdly, some studies had included patients with prior history of bladder cancer, a group associated with the poor prognosis. Lastly, the use of chemotherapy in an adjuvant or neoadjuvant setting could also influence the outcomes. Subgroup analysis, according to a number of adverse pathological factors was also not possible due to lack of data. We were also not able to perform pooled analyses for tumor size as it was reported differently in different studies. Some studies had reported it as a continuous variable and others had reported it as a dichotomous variable with different cut-offs. Most of the studies in this review lack a central review of pathological specimens and have been based on the interpretation of a single pathologist. Furthermore, many of the studies did not properly define various pathological characteristics such as LVI, site of margin positivity, percentage of tumor necrosis and percentage of variant histology in the tumor.

## CONCLUSION

From this review, we noted tumor grade, stage, presence of LVI, lymph node metastasis, hydronephrosis, variant histology, sessile tumors, margin positivity and multifocality were associated with poor RFS, CSS and OS. The presence of CIS was associated with poor RFS and CSS but not OS. Tumor necrosis was associated with the worst CSS and OS but not RFS. Tumor location was not a predictor of any of the survival parameters.

## Figures and Tables

**Supplementary Figure 1:**
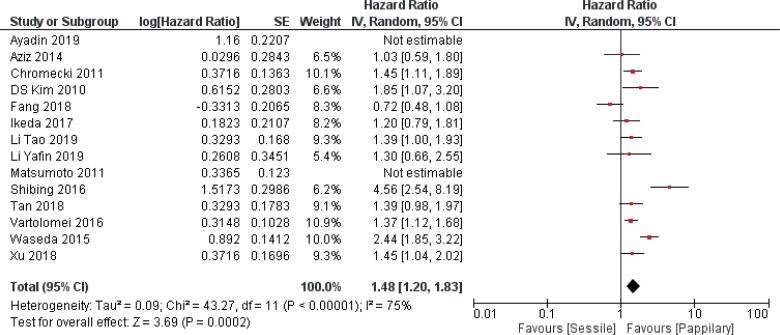
Forest plot depicting RFS for architecture

**Supplementary Figure 2:**
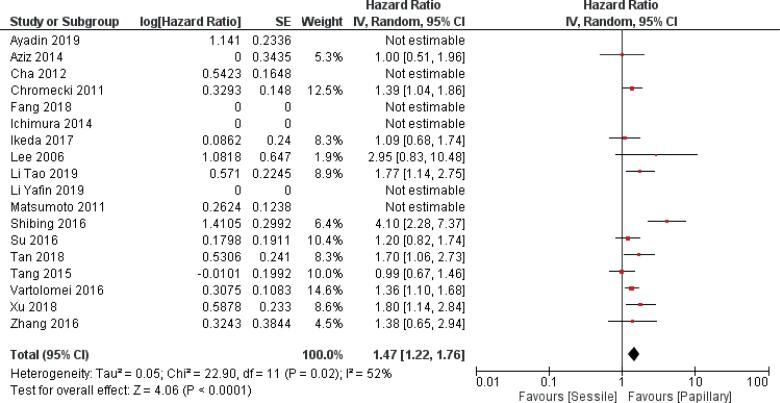
Forest plot depicting CSS for architecture.

**Supplementary Figure 3:**
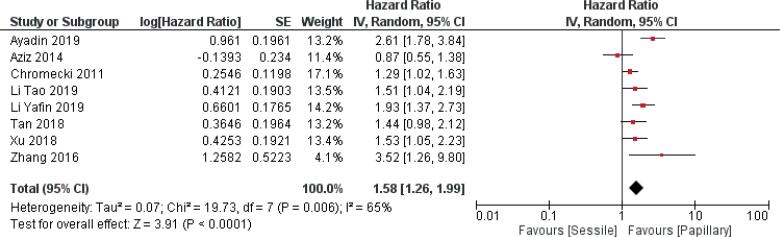
Forest plot depicting OS for architecture.

**Supplementary Figure 4:**
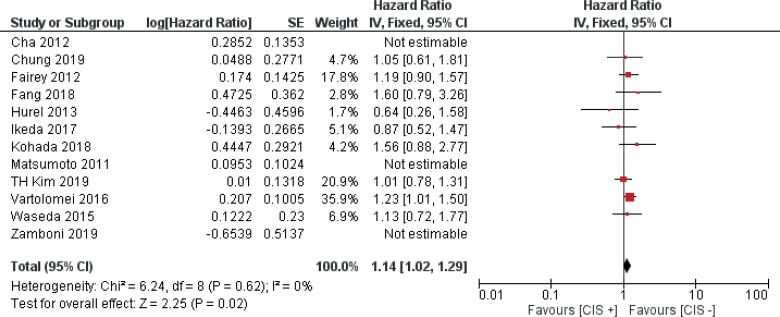
Forest plot depicting RFS for carcinoma in situ.

**Supplementary Figure 5:**
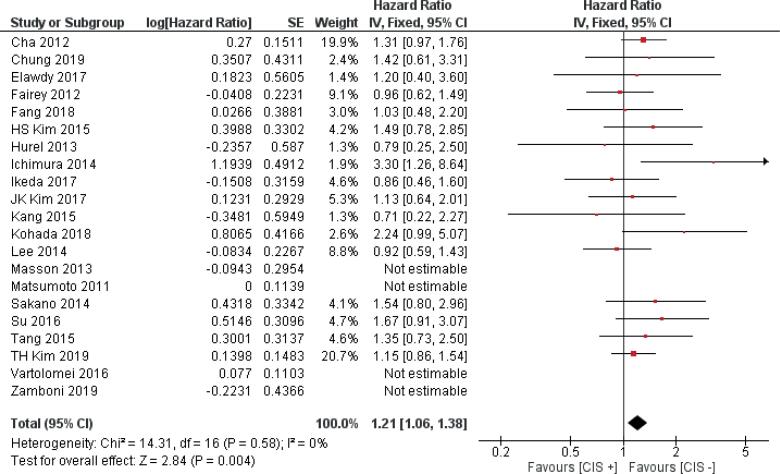
Forest plot depicting CSS for carcinoma in situ.

**Supplementary Figure 6:**
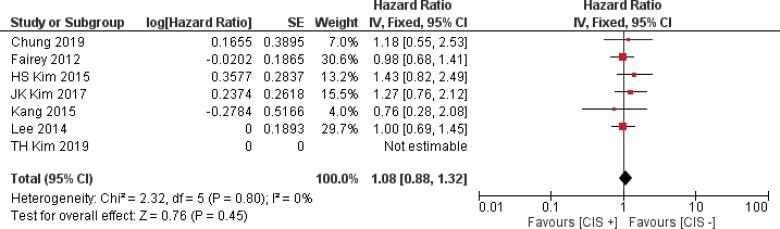
Fore st plot depicting OS for carcinoma in situ.

**Supplementary Figure 7:**
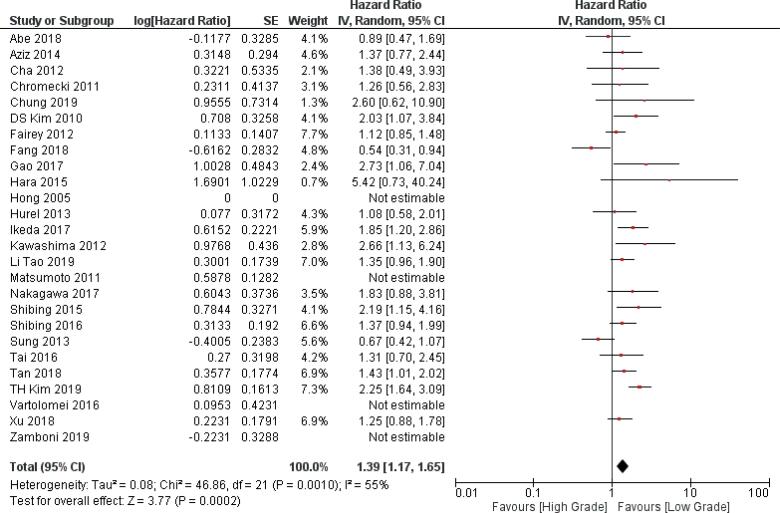
Forest plot depicting RFS for grade.

**Supplementary Figure 8:**
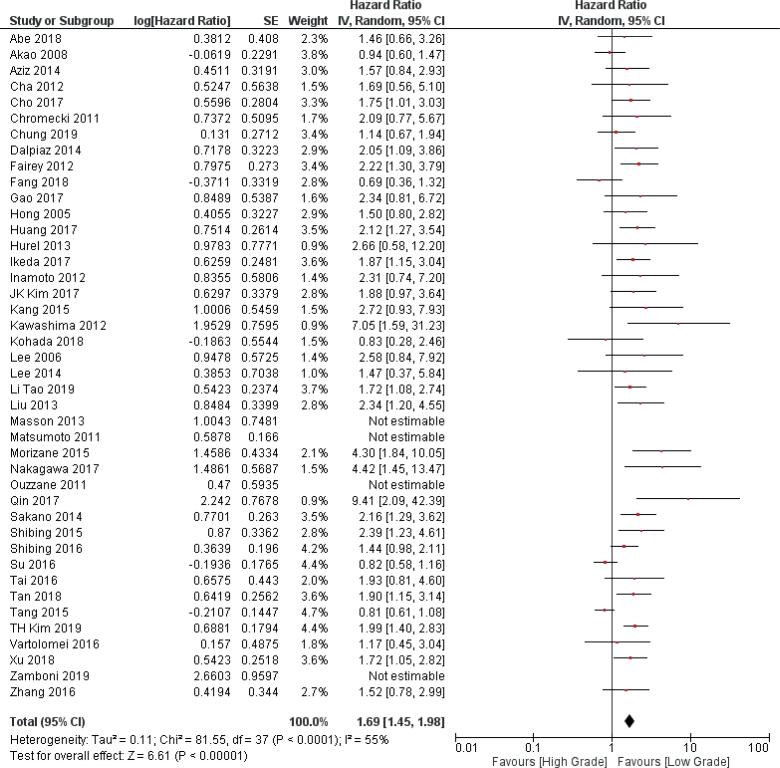
Forest plot depicting CSS for grade.

**Supplementary Figure 9:**
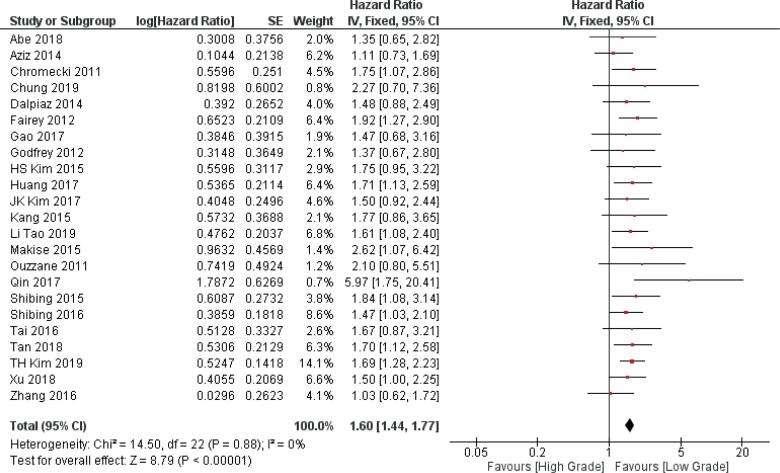
Forest plot depicting OS for grade.

**Supplementary Figure 10:**
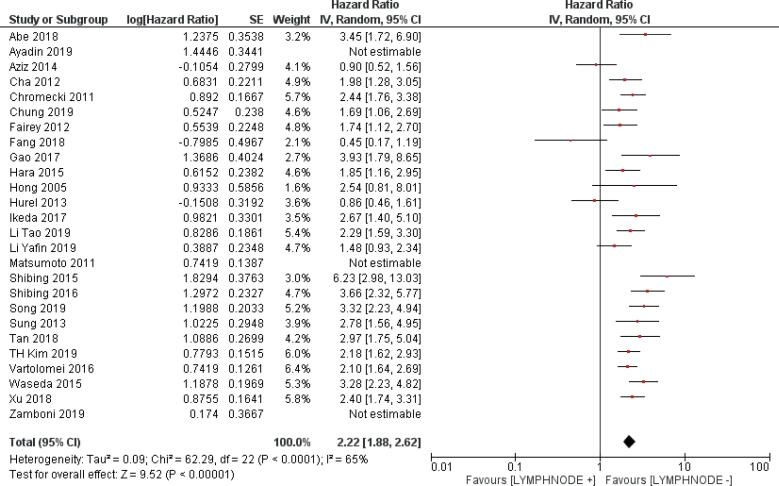
Forest plot depicting RFS for lymph node positivity.

**Supplementary Figure 11:**
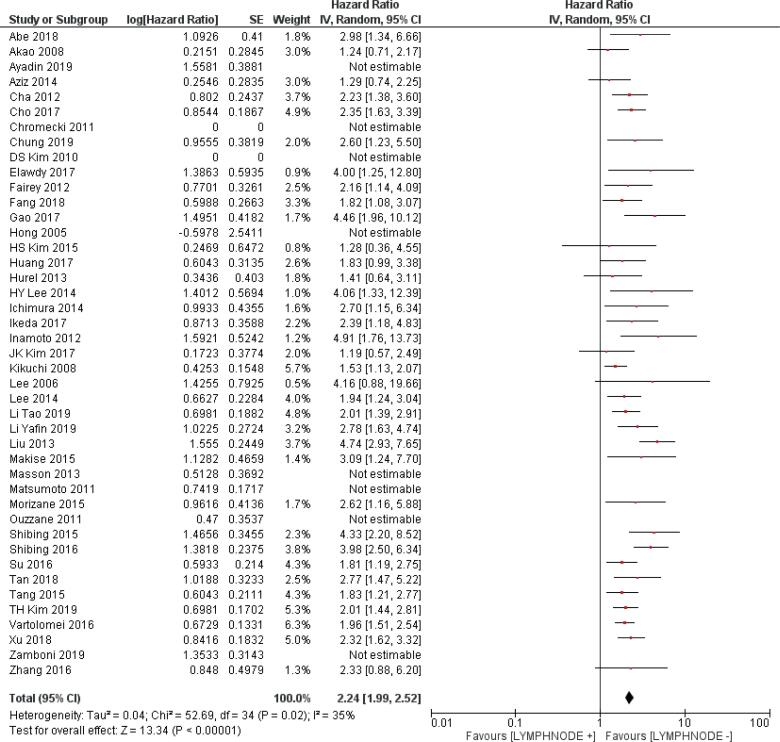
Forest plot depicting CSS for lymph node positivity.

**Supplementary Figure 12:**
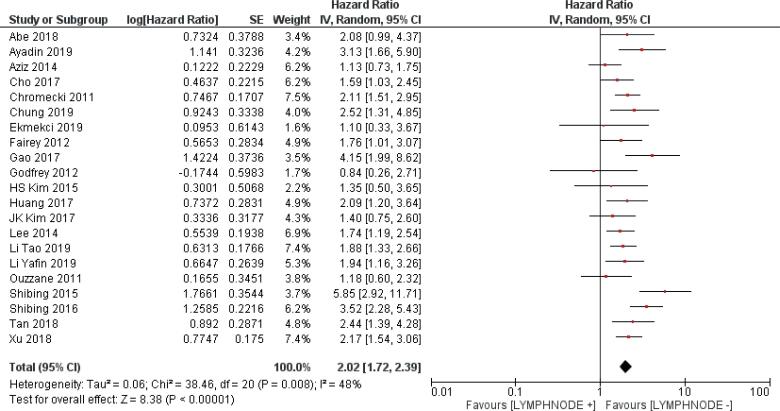
Forest plot depicting OS for lymph node positivity.

**Supplementary Figure 13:**
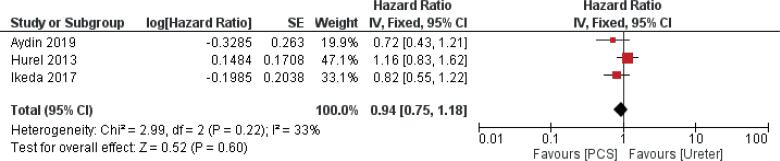
Forest plot depicting RFS for location of tumor.

**Supplementary Figure 14:**
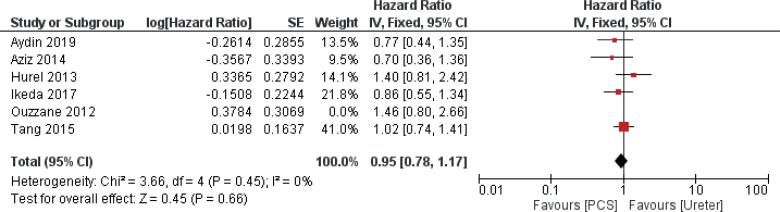
Forest plot depicting CSS for location of tumor.

**Supplementary Figure 15:**
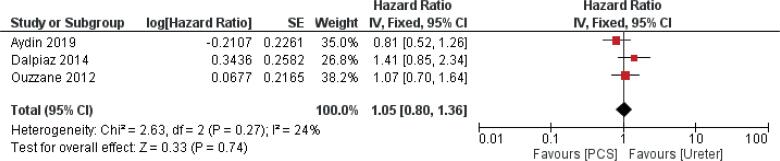
Forest plot depicting OS for location of tumor.

**Supplementary Figure 16:**
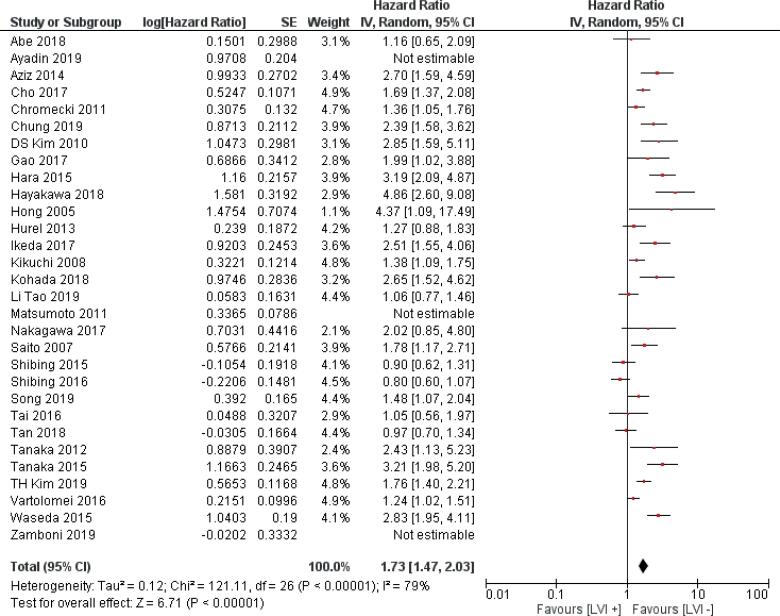
Forest plot depicting RFS for lymphovascular invasion.

**Supplementary Figure 17:**
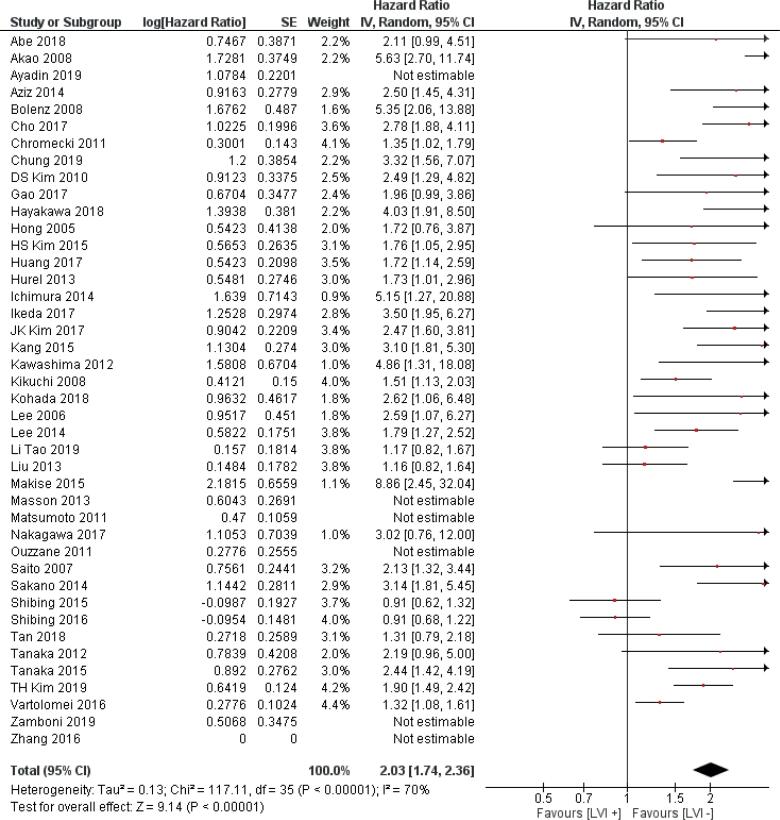
Forest plot depicting CSS for lymphovascular invasion.

**Supplementary Figure 18:**
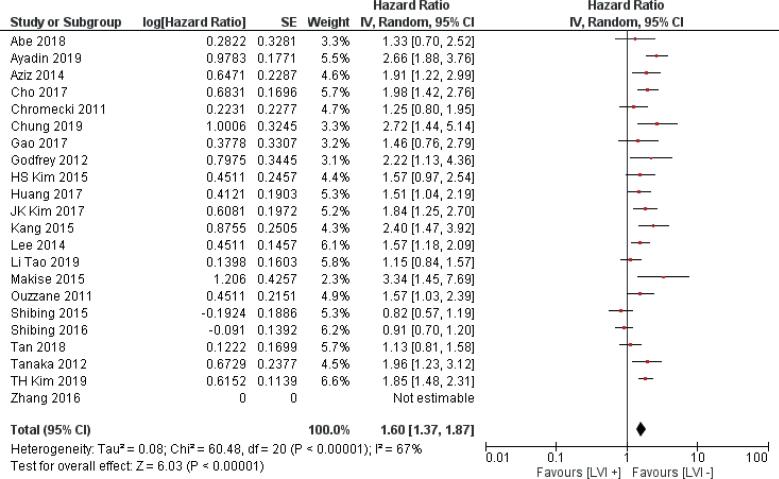
Forest plot depicting OS for lymphovascular invasion.

**Supplementary Figure 19:**
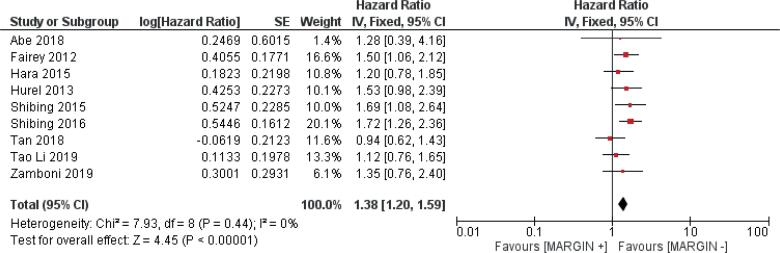
Forest plot depicting RFS for margin positivity.

**Supplementary Figure 20:**
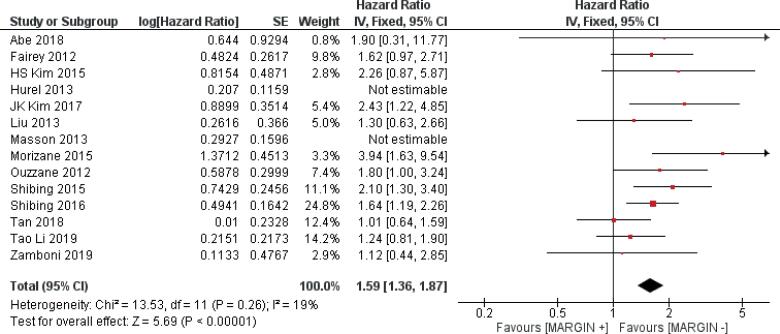
Forest plot depicting CSS for margin positivity.

**Supplementary Figure 21:**
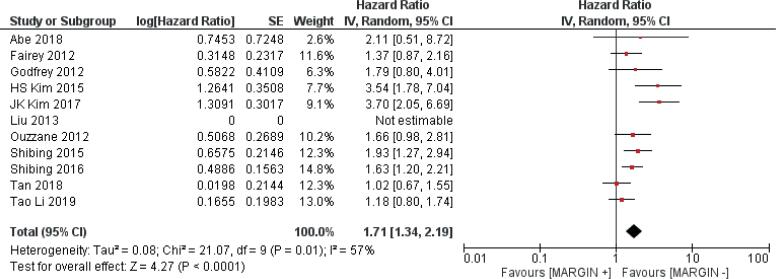
Forest plot depicting OS for margin positivity.

**Supplementary Figure 22:**
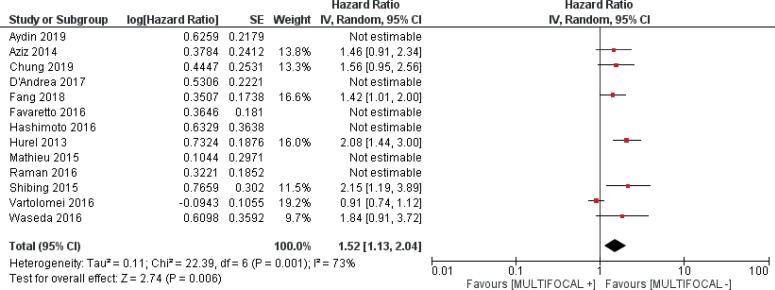
Forest plot depicting RFS for multifocality.

**Supplementary Figure 23:**
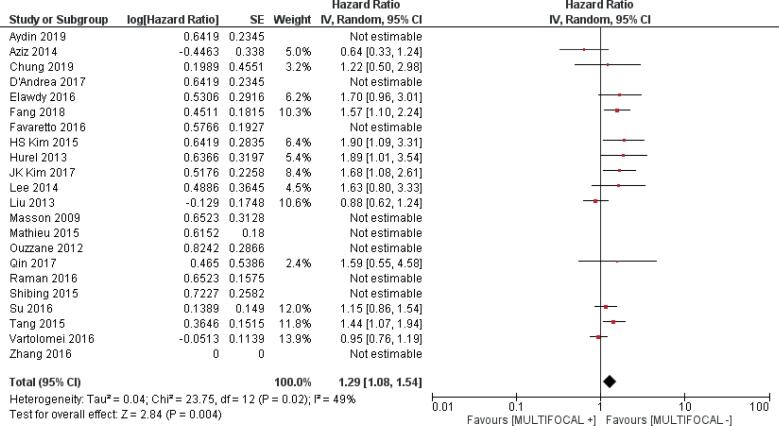
Forest plot depicting CSS for multifocality.

**Supplementary Figure 24:**
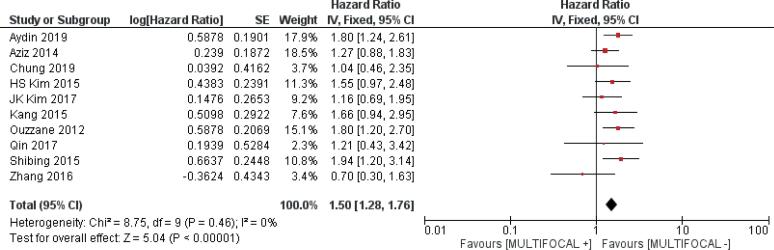
Forest plot depicting OS for multifocality.

**Supplementary Figure 25:**
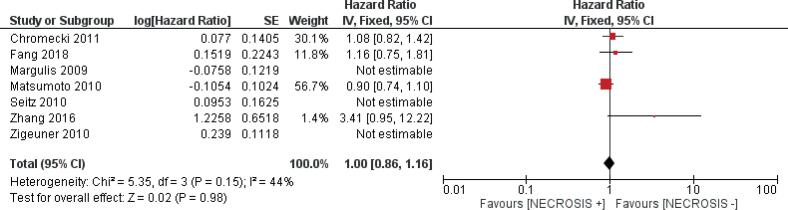
Forest plot depicting RFS for necrosis.

**Supplementary Figure 26:**
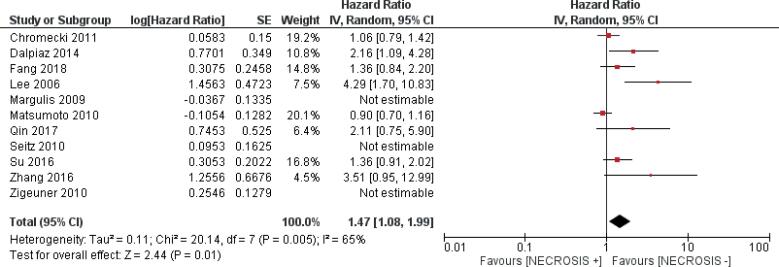
Forest plot depicting CSS for necrosis.

**Supplementary Figure 27:**
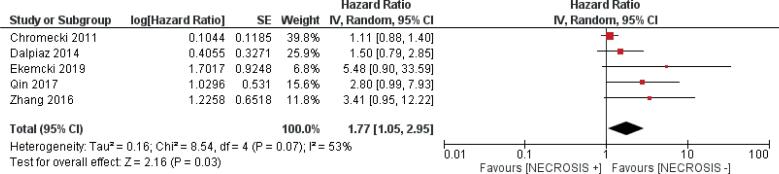
Forest plot depicting OS for necrosis.

**Supplementary Figure 28:**
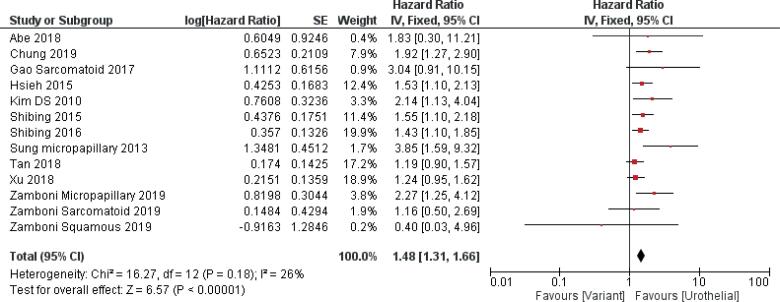
Forest plot depicting RFS for variant histology.

**Supplementary Figure 29:**
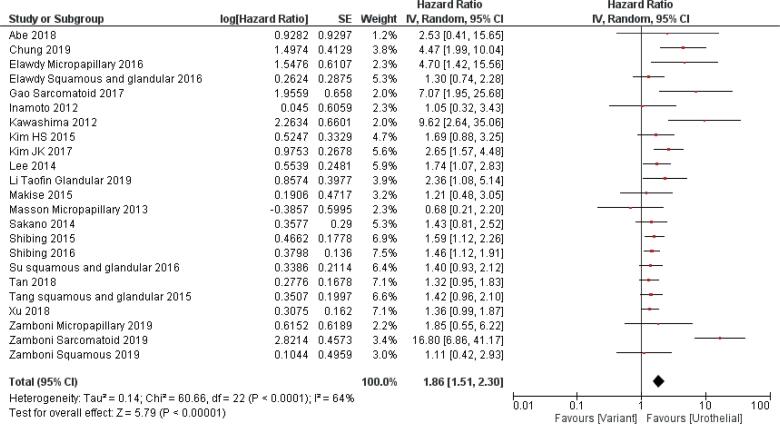
Forest plot depicting CSS for variant histology.

**Supplementary Figure 30:**
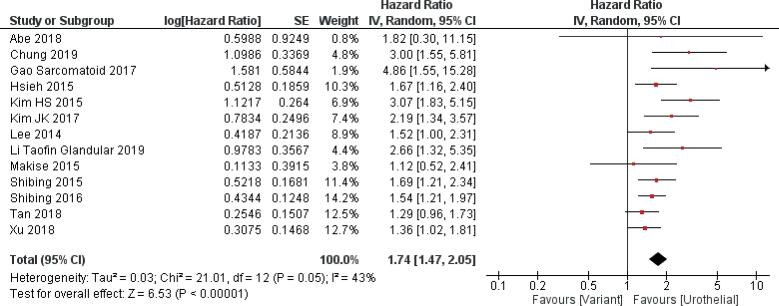
Forest plot depicting OS for variant histology.

**Supplementary Figure 31:**
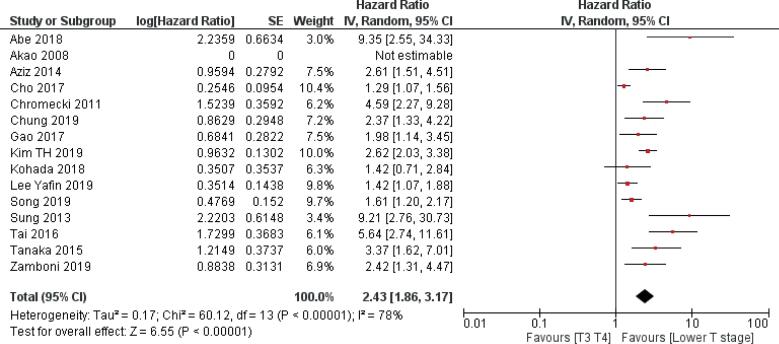
Forest plot depicting RFS for stage.

**Supplementary Figure 32:**
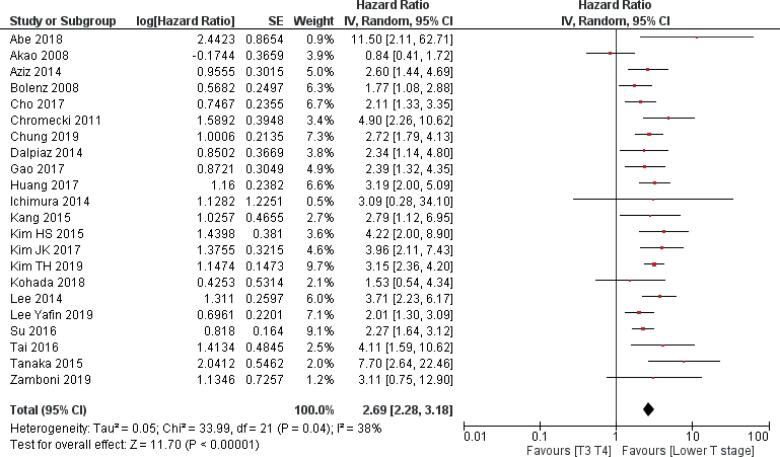
Forest plot depicting CSS for stage.

**Supplementary Figure 33:**
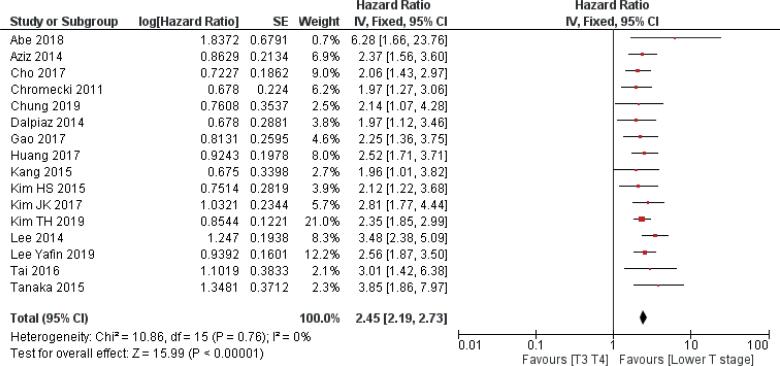
Forest plot depicting OS for stage.

## APPENDIX 1

**Supplementary Table 1:** Pubmed search with search query, search details and results

Query	Search Details	Results
((((Upper tract urothelial carcinoma) OR (Upper tract urothelial cancer)) OR (UTUC)) AND ((((((((((((((( (location)) OR (variant histology)) OR (pathological)) OR (pathology)) OR (multifocality)) OR (sessile)) OR (architecture)) OR (CIS)) OR (carcinoma insitu)) OR (tumor margin)) OR (margin)) OR (tumor necrosis)) OR (LVI)) OR (lymphovascular invasion)) OR (grade)) OR (stage))) AND ((((outcome) OR (survival)) OR (prognostic)) OR (prognosis))	(((((“upper”[All Fields] OR “uppers”[All Fields]) AND ((“tract”[All Fields] OR “tract s”[All Fields]) OR “tracts”[All Fields]) AND ((((“carcinoma, transitional cell”[MeSH Terms] OR ((“carcinoma”[All Fields] AND “transitional”[All Fields]) AND “cell”[All Fields])) OR “transitional cell carcinoma”[All Fields]) OR (“urothelial”[All Fields] AND “carcinoma”[All Fields])) OR “urothelial carcinoma”[All Fields])) OR ((“upper”[All Fields] OR “uppers”[All Fields]) AND ((“tract”[All Fields] OR “tract s”[All Fields]) OR “tracts”[All Fields]) AND “urothelial”[All Fields] AND (((((((((“cancer s”[All Fields] OR “cancerated”[All Fields]) OR “canceration”[All Fields]) OR “cancerization”[All Fields]) OR “cancerized”[All Fields]) OR “cancerous”[All Fields]) OR “neoplasms”[MeSH Terms]) OR “neoplasms”[All Fields]) OR “cancer”[All Fields]) OR “cancers”[All Fields]))) OR “UTUC”[All Fields]) AND (((((((((“locate”[AU Fields] OR “located”[All Fields]) OR “locater”[All Fields]) OR “locates”[All Fields]) OR “locating”[All Fields]) OR “location”[All Fields]) OR “locational”[AU Fields]) OR “locations”[All Fields]) OR “locator”[All Fields]) OR “locators”[All Fields])) OR (((“variant”[All Fields] OR “variant s”[All Fields]) OR “variants”[All Fields]) AND (((((“anatomy and histology”[MeSH Subheading] OR (“anatomy”[All Fields] AND “histology”[All Fields])) OR “anatomy and histology”[All Fields]) OR “histology”[All Fields]) OR “histology”[MeSH Terms]) OR “histologies”[All Fields]))) OR (((((“pathologic”[All Fields] OR “pathologically”[All Fields]) OR “pathologics”[All Fields]) OR “pathology”[MeSH Terms]) OR “pathology”[All Fields]) OR “pathological”[All Fields])) OR (((“pathology”[MeSH Terms] OR “pathology”[All Fields]) OR “pathologies”[All Fields]) OR “pathology”[MeSH Subheading])) OR (((“multifocal”[All Fields] OR “multifocality”[All Fields]) OR “multifocally”[All Fields]) OR “multifocals”[All Fields])) OR “sessile”[All Fields]) OR ((((((“architectural”[All Fields] OR “architecturally”[All Fields]) OR “architecture”[MeSH Terms]) OR “architecture”[All Fields]) OR “architecture s”[All Fields]) OR “architectured”[All Fields]) OR “architectures”[All Fields])) OR “CIS”[All Fields]) OR ((((“carcinoma”[MeSH Terms] OR “carcinoma”[All Fields]) OR “carcinomas”[All Fields]) OR “carcinoma s”[All Fields]) AND “insitu”[All Fields])) OR ((((((((((((((((((((“cysts”[MeSH Terms] OR “cysts”[All Fields]) OR “cyst”[AU Fields]) OR “neoplasm s”[All Fields]) OR “neoplasms”[MeSH Terms]) OR “neoplasms”[All Fields]) OR “neoplasm”[All Fields]) OR “neurofibroma”[MeSH Terms]) OR “neurofibroma”[All Fields]) OR “neurofibromas”[All Fields]) OR “tumor s”[All Fields]) OR “tumoral”[All Fields]) OR “tumorous”[All Fields]) OR “tumour”[All Fields]) OR “tumor”[All Fields]) OR “tumour s”[All Fields]) OR “tumoural”[All Fields]) OR “tumourous”[All Fields]) OR “tumours”[All Fields]) OR “tumors”[All Fields]) AND ((((((((“margin s”[All Fields] OR “marginal”[All Fields]) OR “marginals”[All Fields]) OR “margined”[All Fields]) OR “margins of excision”[MeSH Terms]) OR (“margins”[All Fields] AND “excision”[All Fields])) OR “margins of excision”[All Fields]) OR “margin”[All Fields]) OR “margins”[All Fields]))) OR ((((((((“margin s”[All Fields] OR “marginal”[All Fields]) OR “marginals”[All Fields]) OR “margined”[All Fields]) OR “margins of excision”[MeSH Terms]) OR (“margins”[All Fields] AND “excision”[All Fields])) OR “margins of excision”[All Fields]) OR “margin”[All Fields]) OR “margins”[All Fields])) OR ((((((((((((((((((((“cysts”[MeSH Terms] OR “cysts”[All Fields]) OR “cyst”[All Fields]) OR “neoplasm s”[All Fields]) OR “neoplasms”[MeSH Terms]) OR “neoplasms”[All Fields]) OR “neoplasm”[AU Fields]) OR “neurofibroma”[MeSH Terms]) OR “neurofibroma”[All Fields]) OR “neurofibromas”[All Fields]) OR “tumor s”[All Fields]) OR “tumoral”[All Fields]) OR “tumorous”[All Fields]) OR “tumour”[All Fields]) OR “tumor”[All Fields]) OR “tumour s”[All Fields]) OR “tumoural”[All Fields]) OR “tumourous”[All Fields]) OR “tumours”[All Fields]) OR “tumors”[All Fields]) AND ((((((“necrose”[All Fields] OR “necrosed”[All Fields]) OR “necrosi”[All Fields]) OR “necrosing”[All Fields]) OR “necrosis”[MeSH Terms]) OR “necrosis”[All Fields]) OR “necroses”[All Fields]))) OR “LVI”[All Fields]) OR (“lymphovascular”[All Fields] AND ((((((((“invasibility” [All Fields] OR “invasible”[All Fields]) OR “invasion”[All Fields]) OR “invasions”[All Fields]) OR “invasive”[All Fields]) OR “invasively”[All Fields]) OR “invasiveness”[All Fields]) OR “invasives”[All Fields]) OR “invasivity”[All Fields]))) OR ((((“grade”[All Fields] OR “graded”[All Fields]) OR “grades”[All Fields]) OR “grading”[All Fields]) OR “gradings”[All Fields])) OR ((((“stage”[All Fields] OR “staged”[All Fields]) OR “stages”[All Fields]) OR “staging”[All Fields]) OR “stagings”[All Fields]))) AND ((((“outcome”[All Fields] OR “outcomes”[All Fields]) OR ((((((((((“mortality”[MeSH Subheading] OR “mortality”[All Fields]) OR “survival”[All Fields]) OR “survival”[MeSH Terms]) OR “survivability”[All Fields]) OR “survivable”[All Fields]) OR “survivals”[All Fields]) OR “survive”[All Fields]) OR “survived”[All Fields]) OR “survives”[All Fields]) OR “surviving”[All Fields])) OR (((((((((((“prognostic”[All Fields] OR “prognostical”[All Fields]) OR “prognostically”[All Fields]) OR “prognosticate”[All Fields]) OR “prognosticated”[All Fields]) OR “prognosticates”[All Fields]) OR “prognosticating”[All Fields]) OR “prognostication”[All Fields]) OR “prognostications”[All Fields]) OR “prognosticator”[All Fields]) OR “prognosticators”[All Fields]) OR “prognostics”[All Fields])) OR ((“prognosis”[MeSH Terms] OR “prognosis”[All Fields]) OR “prognoses”[All Fields]))	1,851
((Upper tract urothelial carcinoma) OR (Upper tract urothelial cancer)) OR (UTUC)	(((“upper”[All Fields] OR “uppers”[All Fields]) AND ((“tract”[All Fields] OR “tract s”[All Fields]) OR “tracts”[All Fields]) AND ((((“carcinoma, transitional cell”[MeSH Terms] OR ((“carcinoma”[All Fields] AND “transitional”[All Fields]) AND “cell”[All Fields])) OR “transitional cell carcinoma”[All Fields]) OR (“urothelial”[All Fields] AND “carcinoma”[All Fields])) OR “urothelial carcinoma”[All Fields])) OR ((“upper”[All Fields] OR “uppers”[All Fields]) AND ((“tract”[All Fields] OR “tract s”[All Fields]) OR “tracts”[All Fields]) AND “urothelial”[All Fields] AND (((((((((“cancer s”[All Fields] OR “cancerated”[All Fields]) OR “canceration”[All Fields]) OR “cancerization”[All Fields]) OR “cancerized”[All Fields]) OR “cancerous”[All Fields]) OR “neoplasms”[MeSH Terms]) OR “neoplasms”[All Fields]) OR “cancer”[All Fields]) OR “cancers”[All Fields]))) OR “UTUC”[All Fields]	3,368
((((((((((((((((location)) OR (variant histology)) OR (pathological)) OR (pathology)) OR (multifocality)) OR (sessile)) OR (architecture)) OR (CIS)) OR (carcinoma insitu)) OR (tumor margin)) OR (margin)) OR (tumor necrosis)) OR (LVI)) OR (lymphovascular invasion)) OR (grade)) OR (stage)	(((((((((“locate”[All Fields] OR “located”[All Fields]) OR “locater”[All Fields]) OR “locates”[All Fields]) OR “locating”[All Fields]) OR “location”[All Fields]) OR “locational”[All Fields]) OR “locations”[All Fields]) OR “locator”[All Fields]) OR “locators”[All Fields])) OR (((“variant”[All Fields] OR “variant s”[All Fields]) OR “variants”[All Fields]) AND (((((“anatomy and histology”[MeSH Subheading] OR (“anatomy”[All Fields] AND “histology”[All Fields])) OR “anatomy and histology”[All Fields]) OR “histology”[All Fields]) OR “histology”[MeSH Terms]) OR “histologies”[All Fields]))) OR (((((“pathologic”[All Fields] OR “pathologically”[All Fields]) OR “pathologics”[All Fields]) OR “pathology”[MeSH Terms]) OR “pathology” [All Fields]) OR “pathological”[All Fields])) OR (((“pathology”[MeSH Terms] OR “pathology”[All Fields]) OR “pathologies”[All Fields]) OR “pathology”[MeSH Subheading])) OR (((“multifocal”[All Fields] OR “multifocality”[All Fields]) OR “multifocally”[All Fields]) OR “multifocals” [All Fields])) OR “sessile”[All Fields]) OR ((((((“architectural” [All Fields] OR “architecturally” [All Fields]) OR “architecture” [MeSH Terms]) OR “architecture”[All Fields]) OR “architecture s”[All Fields]) OR “architectured”[All Fields]) OR “architectures”[All Fields])) OR “CIS”[All Fields]) OR ((((“carcinoma”[MeSH Terms] OR “carcinoma”[All Fields]) OR “carcinomas”[All Fields]) OR “carcinoma s”[All Fields]) AND “insitu”[All Fields])) OR ((((((((((((((((((((“cysts”[MeSH Terms] OR “cysts”[All Fields]) OR “cyst”[All Fields]) OR “neoplasm s”[All Fields]) OR “neoplasms”[MeSH Terms]) OR “neoplasms”[All Fields]) OR “neoplasm”[All Fields]) OR “neurofibroma”[MeSH Terms]) OR “neurofibroma”[All Fields]) OR “neurofibromas”[All Fields]) OR “tumor s”[All Fields]) OR “tumoral”[All Fields]) OR “tumorous”[All Fields]) OR “tumour”[All Fields]) OR “tumor”[All Fields]) OR “tumour s”[All Fields]) OR “tumoural”[All Fields]) OR “tumourous”[All Fields]) OR “tumours”[All Fields]) OR “tumors”[All Fields]) AND ((((((((“margin s”[All Fields] OR “marginal”[All Fields]) OR “marginals”[All Fields]) OR “margined”[All Fields]) OR “margins of excision”[MeSH Terms]) OR (“margins”[All Fields] AND “excision”[All Fields])) OR “margins of excision”[All Fields]) OR “margin”[All Fields]) OR “margins”[All Fields]))) OR ((((((((“margin s”[All Fields] OR “marginal”[All Fields]) OR “marginals”[All Fields]) OR “margined”[All Fields]) OR “margins of excision”[MeSH Terms]) OR (“margins”[All Fields] AND “excision”[All Fields])) OR “margins of excision”[All Fields]) OR “margin”[All Fields]) OR “margins”[All Fields])) OR ((((((((((((((((((((“cysts”[MeSH Terms] OR “cysts”[All Fields]) OR “cyst”[All Fields]) OR “neoplasm s”[All Fields]) OR “neoplasms”[MeSH Terms]) OR “neoplasms”[All Fields]) OR “neoplasm”[All Fields]) OR “neurofibroma”[MeSH Terms]) OR “neurofibroma”[All Fields]) OR “neurofibromas”[All Fields]) OR “tumor s”[All Fields]) OR “tumoral”[All Fields]) OR “tumorous”[All Fields]) OR “tumour”[All Fields]) OR “tumor”[All Fields]) OR “tumour s”[All Fields]) OR “tumoural”[All Fields]) OR “tumourous”[All Fields]) OR “tumours”[All Fields]) OR “tumors”[All Fields]) AND ((((((“necrose”[All Fields] OR “necrosed”[All Fields]) OR “necrosi”[All Fields]) OR “necrosing”[All Fields]) OR “necrosis”[MeSH Terms]) OR “necrosis”[All Fields]) OR “necroses”[All Fields]))) OR “LVI”[All Fields]) OR (“lymphovascular”[All Fields] AND ((((((((“invasibility”[All Fields] OR “invasible”[All Fields]) OR “invasion”[All Fields]) OR “invasions”[All Fields]) OR “invasive”[All Fields]) OR “invasively”[All Fields]) OR “invasiveness”[All Fields]) OR “invasives”[All Fields]) OR “invasivity”[All Fields]))) OR ((((“grade”[All Fields] OR “graded”[All Fields]) OR “grades”[All Fields]) OR “grading”[All Fields]) OR “gradings”[All Fields])) OR ((((“stage”[All Fields] OR “staged”[All Fields]) OR “stages”[All Fields]) OR “staging”[All Fields]) OR “stagings”[All Fields])	6,005,790
(((outcome) OR (survival)) OR (prognostic)) OR (prognosis)	“outcome”[All Fields] OR “outcomes”[All Fields] OR “mortality”[MeSH Subheading] OR “mortality”[All Fields] OR “survival”[All Fields] OR “survival”[MeSH Terms] OR “survivability”[All Fields] OR “survivable”[All Fields] OR “survivals”[All Fields] OR “survive”[All Fields] OR “survived”[All Fields] OR “survives”[All Fields] OR “surviving”[All Fields] OR “prognostic”[All Fields] OR “prognostical”[All Fields] OR “prognostically”[All Fields] OR “prognosticate”[All Fields] OR “prognosticated”[All Fields] OR “prognosticates”[All Fields] OR “prognosticating”[All Fields] OR “prognostication”[All Fields] OR “prognostications”[All Fields] OR “prognosticator”[All Fields] OR “prognosticators”[All Fields] OR “prognostics” [All Fields] OR “prognosis”[MeSH Terms] OR “prosnosis”[All Fields] OR “prosnoses”[All Fields]	4,432,884
outcome	“outcome”[All Fields] OR “outcomes”[All Fields]	2,461,422
survival	“mortality”[MeSH Subheading] OR “mortality”[All Fields] OR “survival”[All Fields] OR “survival”[MeSH Terms] OR “survivability”[All Fields] OR “survivable”[All Fields] OR “survivals”[All Fields] OR “survive”[All Fields] OR “survived”[All Fields] OR “survives”[All Fields] OR “surviving”[All Fields]	2,086,064
prognostic	“prognostic”[All Fields] OR “prognostical”[All Fields] OR “prognostically”[All Fields] OR “prognosticate”[All Fields] OR “prognosticated” [All Fields] OR “prognosticates” [All Fields] OR “prognosticating” [All Fields] OR “prognostication”[All Fields] OR “prognostications”[All Fields] OR “prognosticator”[All Fields] OR “prognosticators”[All Fields] OR “prosnostics”[All Fields]	301,748
prognosis	“prognosis”[MeSH Terms] OR “prognosis”[All Fields] OR “prognoses”[All Fields]	1,823,869
location	“locate”[All Fields] OR “located”[All Fields] OR “locater”[All Fields] OR “locates”[All Fields] OR “locating”[All Fields] OR “location”[All Fields] OR “locational”[All Fields] OR “locations”[All Fields] OR “locator”[All Fields] OR “locators”[All Fields]	771,575
variant histology	((“variant”[All Fields] OR “variant s”[All Fields]) OR “variants”[All Fields]) AND (((((“anatomy and histology”[MeSH Subheading] OR (“anatomy”[All Fields] AND “histology”[All Fields])) OR “anatomy and histolosy”[All Fields]) OR “histolosy”[All Fields]) OR “histolosy”[MeSH Terms]) OR “histolosies”[All Fields])	74,389
pathological	“pathologic”[All Fields] OR “pathologically”[All Fields] OR “pathologics”[All Fields] OR “pathology”[MeSH Terms] OR “pathology”[All Fields] OR “pathological”[All Fields]	3,795,533
pathology	“pathology”[MeSH Terms] OR “pathology”[All Fields] OR “pathologies”[All Fields] OR “pathology”[MeSH Subheading]	3,554,131
multifocality	“multifocal”[All Fields] OR “multifocality”[All Fields] OR “multifocally”[All Fields] OR “multifocals”[All Fields]	33,181
Sessile	“sessile”[All Fields]	7,165
architecture	“architectural”[All Fields] OR “architecturally”[All Fields] OR “architecture”[MeSH Terms] OR “architecture”[All Fields] OR “architecture s”[All Fields] OR “architectured”[All Fields] OR “architectures”[All Fields]	171,172
CIS	“CIS”[All Fields]	123,073
carcinoma insitu	(((“carcinoma”[MeSH Terms] OR “carcinoma”[All Fields]) OR “carcinomas”[All Fields]) OR “carcinoma s”[All Fields]) AND “insitu”[All Fields]	1,315
tumor margin	(((((((((((((((((((“cysts”[MeSH Terms] OR “cysts”[All Fields]) OR “cyst”[All Fields]) OR “neoplasm s”[All Fields]) OR “neoplasms”[MeSH Terms]) OR “neoplasms”[All Fields]) OR “neoplasm”[All Fields]) OR “neurofibroma”[MeSH Terms]) OR “neurofibroma”[All Fields]) OR “neurofibromas”[All Fields]) OR “tumor s”[All Fields]) OR “tumoral”[All Fields]) OR “tumorous”[All Fields]) OR “tumour”[All Fields]) OR “tumor”[All Fields]) OR “tumour s”[All Fields]) OR “tumoural”[All Fields]) OR “tumourous”[All Fields]) OR “tumours”[All Fields]) OR “tumors”[All Fields]) AND ((((((((“margin s”[All Fields] OR “marginal”[All Fields]) OR “marginals”[All Fields]) OR “margined”[All Fields]) OR “margins of excision”[MeSH Terms]) OR (“margins”[All Fields] AND “excision”[All Fields])) OR “margins of excision”[All Fields]) OR “margin”[All Fields]) OR “marsins”[All Fields])	63,557
Margin	(((((((“margin s”[All Fields] OR “marginal”[All Fields]) OR “marginals”[All Fields]) OR “margined”[All Fields]) OR “margins of excision”[MeSH Terms]) OR (“margins”[All Fields] AND “excision”[All Fields])) OR “margins of excision”[All Fields]) OR “marsin”[All Fields]) OR “marsins”[All Fields]	159,816
tumor necrosis	(((((((((((((((((((“cysts”[MeSH Terms] OR “cysts”[All Fields]) OR “cyst”[All Fields]) OR “neoplasm s”[All Fields]) OR “neoplasms”[MeSH Terms]) OR “neoplasms”[All Fields]) OR “neoplasm”[All Fields]) OR “neurofibroma”[MeSH Terms]) OR “neurofibroma”[All Fields]) OR “neurofibromas”[All Fields]) OR “tumor s”[All Fields]) OR “tumoral”[All Fields]) OR “tumorous”[All Fields]) OR “tumour”[All Fields]) OR “tumor”[All Fields]) OR “tumour s”[All Fields]) OR “tumoural”[All Fields]) OR “tumourous”[All Fields]) OR “tumours”[All Fields]) OR “tumors”[All Fields]) AND ((((((“necrose”[All Fields] OR “necrosed”[All Fields]) OR “necrosi”[All Fields]) OR “necrosing”[All Fields]) OR “necrosis”[MeSH Terms]) OR “necrosis”[All Fields]) OR “necroses”[All Fields])	254,227
LVI	“LVI”[All Fields]	1,509
lymphovascular invasion	“lymphovascular”[All Fields] AND ((((((((“invasibility”[All Fields] OR “invasible”[All Fields]) OR “invasion”[All Fields]) OR “invasions”[All Fields]) OR “invasive”[All Fields]) OR “invasively”[All Fields]) OR “invasiveness”[All Fields]) OR “invasives”[All Fields]) OR “invasivity”[All Fields])	5,770
Grade	“grade”[All Fields] OR “graded”[All Fields] OR “grades”[All Fields] OR “grading”[All Fields] OR “gradings”[All Fields]	451,054
Stage	“stage”[All Fields] OR “staged”[All Fields] OR “stages”[All Fields] OR “staging”[All Fields] OR “stagings”[All Fields]	1,203,520
UTUC	“UTUC”[All Fields]	869
Upper tract urothelial cancer	(“upper”[All Fields] OR “uppers”[All Fields]) AND ((“tract”[All Fields] OR “tract s”[All Fields]) OR “tracts”[All Fields]) AND “urothelial”[All Fields] AND (((((((((“cancer s”[All Fields] OR “cancerated”[All Fields]) OR “canceration”[All Fields]) OR “cancerization”[Ali Fields]) OR “cancerized”[All Fields]) OR “cancerous”[All Fields]) OR “neoplasms”[MeSH Terms]) OR “neoplasms”[All Fields]) OR “cancer”[All Fields]) OR “cancers”[All Fields])	2,343
Upper tract urothelial carcinoma	(“upper”[All Fields] OR “uppers”[All Fields]) AND ((“tract”[All Fields] OR “tract s”[All Fields]) OR “tracts”[All Fields]) AND ((((“carcinoma, transitional cell”[MeSH Terms] OR ((“carcinoma”[All Fields] AND “transitionar[All Fields]) AND “cell”[All Fields])) OR “transitional cell carcinoma”[All Fields]) OR (“mothelial”[All Fields] AND “cell”[All Fields])) OR “urothelial carcinoma”[All Fields])	3,098

**Supplementary File S3:** Characteristics of included studies.

S. no	Author Year Country	Number of patients	Study type	Multicentre (Yes/ No)	Age (Mean/ Media n (range)	Male/ Female	Surgery (O/L)	L. Node Dissection	Pathological Stage (pTais/pT1/pT2/pT3/pT4)	LVI (PRESENT)	No. of Patient with Necrosis	Definition of necrosis	Tumor Site	Tumor Grade (1,2/3/unknown)	Adjuvant Chemotherapy (Yes/No)	Variant Histology (%)	Duration of Follow up	Parameters controlled in multivariate analysis	Survival outcomes assessed	NOS
1.	Hayakawa 2017 Japan	181	R	N	73 (36-93)	140/41	NA	N	<T2-78>T2-103	79	NA	NA	P-101 U-70 Both-10	LG-52 HG-129	NA	30	53 (1-253)	-LVI-PD-1 expression in tumor nest	CSS PFS	6
2.	Hong 2005 Korea	73	R	N	59.1	NA	NA	Y-37	Ta-15 T1-18 T2-9 T3-27 T4-4	18	NA	NA	P-40 U-33	G1-6 G2-35 G3-32	13	NA	42.3	-LVI - grade -stage	DSS RFS	6
3.	Hsieh 2015 Taiwan	206	R	N	63 (22-84)	138/68	NA	NA	NA	NA	NA	NA	Upper urinarytract-119 Bladder-84 Both-3		206	53	134.5	-Histopathological Variant -Renal function -Visceral metastasis	OS PFS OS	6
4.	Hurel 2013 France	551	R	Y	69.4 (61.8-76.4)	365/186	O-551	Y	Ta/Tis-142 T1-124 T2-53 T3-193 T4-39	163	NA	NA	P-302 U-169 Both-80	G1-80 G2-251 G3-415	79	NA	26.8 (10.348.7)	-Multifocal -pT3 stage -LVI -positive surgical margin(MFS)	CSS RFS MFS	6
5.	Ichimura 2014 Japan	171	R	N	70	119/52	NA	Y	Ta/Tis-44 T1-31 T2-18 T3-69 T4-9	74	NA	NA	P-103 U-68	LG-19 HG-152	NA	NA	56	-High CD204* -LVI -LN Mets	RFS MFS CSS	6
6.	Ike da 2017 Japan	441	R	Y	69 (62-75)	319/122	O-247 L-194	Y	Ta/Tis-86 T1-92 T2-81 T3-158 T4-24	156	NA	NA	P-245 U-196	G1/2-305 G3-130	100	37	35.7	-T stage - Lymph node status -Grade3 -LVI -positive STSM	DFS CSS	7
7.	Kang 2015 Korea	440	R	Y	NA	305/135	NA	Y	Ta/Tis-31 T1-135 T2-101 T3-155 T4-8	76	NA	NA	P-159 U-219 Both-62	LG-110 HG-330	78	NA	31 (15-57)	-Locally advanced stage -Node positive status -LVI -Margin status	OS DSS	8
8.	Kim DS 2010 Korea	238	R	N	64.1 (25-91)	164/74	NA	Y	Ta-T2-131 T3-107	31	NA	NA	P-134 U-104	LG-95 HG-143	NA	24	53.4 (3-240)	-Tumor architecture -squamous differentiation -LVI -Tumor grade	RFS CSS	7
9.	Kim JK 2017 Korea	452	R	N	64±10.2	347/105	O-332 L-120	Y	T0/a/is/1-187 T2-75 T3/4-188	99	NA	NA	P-223 U-165 Both-64	LG-59 HG-81	110	41	67.8 (0-254)	-Age -T stage - multifocality -Positive STSM -tumor location -variant histology -LVI	OS CSS	7
10.	Kim SH 2015 Korea	371	R	N	64.7 (57.7)	287/84	O-271 L-100	Y	pT0/a/is/1-162 pT2-63 pT3/4-146	71	NA	NA	P-183 Ur40 Both-48	LG-125 HG-246	85	28	50.8	-LRUN - stage -grade	OS CSS	7
11.	Lee Sang 2006 Korea	119	R	N	62 (36-90)	92/27	NA	Y	Ta/T1-38 pT2-4-81	30	19	>10% macroscopic necrosis	P-54 U-65	G1/2-76 G3-43	40	NA	41 (2-164)	-T stage -LVI -Tumor necrosis	DSS	7
12.	Lee Young 2014 Korea	341	R	N	63.1 (56.4-70.5)	301/40	NA	Y	Ta/Tis-54 T1-81 T2-58 T3-144 T4-4	70	NA	NA	NA	G1-39 G2-206 G3-96	86	27	66.8 (30-95.3)	-Age -T stage -LVI -positive STSM -Nodal metastasis -Histological variant	CSS OS	7
13.	Lee Hsiang 2014 Taiwan	250	R	N	68	108/142	O-166 L-84	Y	Ta/Tis-40 T1-53 T2-73 T3-70 T4-14	60	NA	NA	P-128 U-122	LG-55 HG-195	42	NA	41	-T stage - Lymph node involvement -LVI -Concomitant bladder tumor(RFS)	CSS MFS RFS	7
14.	LI Tao 2019 China	704	R	N	66±11.4	401/303	O-474 L-230	Y	</=T2-359 >/=T3-345	107	NA	NA	P-375 U-202 Both-127	LG-185 HG-519	286	162	39 (34-43)	-Low lymphocyte to monocyte ratio -Tumor size >/=3cm -High tumor grade -Advance tumor stage(>/=T3) -Lymph node invasion -Tumor architecture -Concomitant variant histology -Albumin to globulin ratio	CSS RFS OS	7
15.	LI Yifan 2019 China	602	R	N	66.77±9.90	285/317	NA	Y	Ta-6 T1-322 T2-2956T3-238 T4-24	46	114	NA	P-310 U-292	G1-15 G2-342 G3-245	NA	105	6138-102)	-High AST/ALT -T stage -N stage -Age -Gender -Tumor location -Tumor size -Glandular differentiation	CSS OS RFS	7
16.	Liu 2013 China	421	R	Y	62 (51-70)	285/136	O-364 L-57	Y	Ta/Tis/T1-157 T2-91 T3-144 T4-29	101	NA	NA	P-225 U-196	G1-87 G2-128 G3-206	88	NA	NA	-Female gender -LVI -Tumor grade -Tumor stage - N stage	CSS	6
17.	Masson 2013 France	519	R	Y	68.4 (61.2-76.5)	342/177	O-519	Y	Ta/is/1246 pT2/3/4-273	361	NA	NA	P-289 U-154 Both-76	G1-46 G2-167 G3-306	80	39	27 (10.2-48.7)	-T stage -LVI -margin status -Adjuvant chemotherapy	CSS MFS	6
18.	Matsumoto 2011 Japan	2163	R	Y	69 (61-76)	1478/685	O-1790 L-373	Y	T0-10 Ta-450 Tis-36 T1-488 T2-401 T3-667 T4-111	481	496	NA	NA	LG-655 HG-1508	224	NA	36 (15.3-71.1)	-Age -T stage -Tumor grade -LVI -Tumor architecture -N stage	RFS CSS	7
19.	Nakagawa 2017 Japan	109	R	Y	71 (64-77)	67/42	NA	Y	T3-104 T4-5	78	NA	NA	P-50 U-23 Both-36	G1-0 G2-40 G3-69	43	NA	46.5 (23.2-76.7)	-Adjuvant chemotherapy lower nuclear grade -absence of hydronephrosis	RFS CSS	8
20.	Ouzzane 2012 France	714	R	Y	70 (60-75)	484/228	NA	Y	Ta/Tis-131 T1-216 T2-124 T3-205 T4-40	157	NA	NA	P-388 U-236 Both-90	G1-71 G2-244 G3-399	NA	NA	27 (10-50)	-Age -T stage - surgical margin	CSS MFS OS	6
21.	Qin 2017 China	346	R	N	66.61± 9.897	206/140	NA	N	Ta/is/1258 pT2/3/4-88	NA	18	NA	P-175 U-171	LG-59 HG-287	169	50	21 (10-36)	-T stage -Tumor grade -variant histology -adjuvant chemotherapy	RFS CSS OS	6
22.	Kikuchi 2009 japan	1453	R	Y	69.7 (27-97)	986/467	NA	Y	Ta-295 Tis-28 T1-317 T2-269 T3-475 T4-69	349	387	NA	P-958 U-495	LG-516 HG-937	169	NA	NA	-T stage -Tumor grade -N stage -LVI	CSS RFS	6
23.	Kawashima 2011 Japan	93	R	Y	NA	68/25	NA	Y	>T3-93	54	NA	NA	P-55 U-38	G1-6 G2-31 G3-56	38	11	NA	-Adjuvant chemotherapy -Tumor grade -LVI -Sex -Histology	CSS RFS	6
24.	Kim TH 2019 South Korea	1521	R	Y	65 (57-72)	1127/394	O-906 L-615	Y	Ta/Tis-235 T1-404 T2-255 T3-592 T4-35	332	NA	NA	P-682 U-565 Both-274	LG-485 HG-993 Missing-43	340	NA	54.9 (32.7-89.7)	-Previous bladder Tumor -Concomitant bladder tumor -Age -T stage -Tumor grade -LVI -Concomitant CIS -N stage	IVRFS PFS CSS OS	6
25.	Kohada 2018 Japan	148	R	N	71 (64-78)	112/36	NA	Y	Ta/1/2-82 T3/4-66	55	NA	NA	P-82 U-66	G1/2-60 G3-88	25	NA	35.5 (12-66)	-Elevated pre-op Neutrophll- lymphocytes ratio -Hydronephrosis -LVI	CSS RFS	7
26.	Morizane 2015 Japan	345	R	Y	74 (38-95)	234/111	O-244 L-101	Y	<T3-188>/=T3-152	102	NA	NA	P-140 U-205	Non G3- 222 G3-109	80 (23.2%)	29	39.9 (6.1-160)	-ECOG performance status -Number of tumor foci -Serum HB -eGFR -T stage -Histological variant -Tumor grade -Positive LN -INF -LVI -Positive margin	CSS	6
27.	Makise 2015 Japan	140	R	N	NA	101/39	NA	Y	Ta/Tis-36 T1-25 T2-11 T3-60 T4-8	61	NA	NA	P-89 U-51	G1/2-63 G3-77	42	23	NA	-T stage -N stage -LVI -Tumor grade -Age	MFS CSS OS	7
28.	Zhang 2016 China	184	R	N	70 (60-74)	84/100	O-125 L-59	Y	Ta/1-73 T2/3/4-111	28	30	NA	P-99 U-85	G1/2-117 G3-67	NA	NA	78 (34-92)	-preoperative plasma fibrinogen level -Gender -T stage -Age>70 -Preoperative CKD4/5	OS CSS	7
29.	Su 2016 China	687	R	N	<3cm-69 (20-90)>3cm-68 (29-86)	306/381	O-220 L-467	Y	Ta/is/1-129 T2-242 T3-197 T4-19	NA	79	NA	P-380 U-307	G1-21 G2-368 G3-298	NA	81	65 (3-144)	-Older age -Male -presence of hydronephrosis -Advance T stage -Positive LN -preoperative ureteroscopy -Lower tumor grade -N0 status -Tumor multifocality	CSS RFS	7
30.	Huang 2016 China	481	R	N	65.8±11.1	311/170	O-318 L-163	Y	Ta/1-248 T2/3/4-233	76	NA	NA	P-232 U-160 Multifocal-89	LG-163 HG-318	96	NA	40 (24-64)	-F-PLR score -Age >65 -Tumor multifocality -T stage -Higher grade -LVI Higher pN stage	OS CSS	6
31.	Abe 2018 Japan	214	R	Y	70.5 (35-93)	151/63	0-100 L-114	Y 214	42/48/41/75/8	96	NA	NA	P-127 U-82 Both-5	100/113/	14/200	NA	15	-T stage -LVI -Tumor number	RFS CSF OS	7
32.	Akao 2008 Japan	90	R	N	NA	57/33	NA	NA	0/3/24/14/43/6	34	NA	NA	P-51 U-39	4/56/29	24/61	NA	42 (2-179)	-LVI -pT -pN - Tumor grade -Adjuvant therpy	DSS	6
33.	Aydin 2019 USA	348	R	Y	70 (64-77)	163/185	NA	Yes (n=86)	31/103/57/129/28	98	62	NA	P-267 U-81	NA	NA	NA	36	-T stage - LVI -Necrosis- Architecture	RFS CSS OS	7
34.	Aziz 2014 Germany	265	R	Y	67.7 ± 9.85; 69.8 ± 8.85	169/96	NA	Yes (n=59)	106 (Ta-T1)/49/102/8	52	NA	NA	P- 57 U- 33 Both- 26	43/60/162	46/219	NA	37 (9-48)	-ECOG -Tumor multifocality -LN involvement -LVI	RFS DSS ACS	6
35.	Bolenz 2008 Germany	116	R	N	NA	80/36	0-107 L-09	Y 27	9/3/23/28/42/11/20	36	17	10%	P-84 U-32	12/58/46	NA	NA	38	-LVI -Pathological stage	DSS	7
36.	Cha 2012 USA	2244	R	Y	69 (61.6-76.0)	1502/742	NA	Y-129 N-540 X-1575	516/46/537/444/606/80	484	NA	NA	P- 1449 U- 795	HG- 1838 LG- 406	NA	NA	45	-T stage -LN status -LVI -Architecture -CIS	RFS CSM CSS	7
37.	Cho 2017 Korea	1049	R	Y	68.5 (60.5-74.3)	759/290	NA	505	106/316/201/403/23	202	NA	NA	P-489 U-306 Both-92	HG-745 LG-304	Y-300	NA	40 (18.4-64.8)	-T stage -N1 disease -Hydronephrosis -De Ret is Ratio	RFS CSS OS	8
38.	Chromecki 2011 USA	1169	R	Y	69 (3092)	785/384	O-1014 L-155	Y 398	285/20/274/231/318/53	259	287	NA	P-742 U-427	LG-179 HG-982	Y-78	NA	37 (1-197)	-Age -Stage -Grade -Architecture -Necrosis -LVI	CSD OS	7
39.	Chung 2019 Korea	1173	R	Y	68.8 (61-74.6)	849/324	NA	540	Tis/Ta/T1-460 T2-230 T3/T4-483	236	NA	NA	P-542 U-537 Both-94	LG-343 HG-830	Y-357	93 (7.9%)	NA	-Preoperative anemia -HDN -LVI -VH	RFS CSS OS	7
40.	Dalpiaz 2014 Austria	171	R	N	69 +/− 10.1	107/64	NA	NA	T1-79 T2-4=92	NA	21	NA	P-95 U-76	G1-2=92 G3-4=79	NA	NA	31 (13-69)	-p stage -Grade pHistological - Tumor necrosis	CSS OS	7
41.	Ekmekci 2019 Turkey	74	R	Y	63.3 (40-84)	60/14	NA	64	pTa-1613/04/28/13	25	29	NA	P-38 U-7 Both-29	NA	NA	22 (39.2%)	43.5 +/− 48.7	-Tumor necrosis -Tumor differentiation -LN metastasis	DFS OS	7
42.	Elawddy 2016 Osman	305	R	N	59 +/− 11	262/43	O-268 L-24 Renalsparing-13	NA	T0-3 Ta,is.1-196 T2-44 T3-61 T4-1	NA	NA	NA	P-183 U-182	G0-3 G1-16 G2-195 G3-100	NA	NA	34 (6-300)	-Tumor stage -Micropapillary variant	CSS OS	7
43.	Fairey 2012 Canada	849	R	Y	70.5		O-403 L-446	245	<=T1-186 T2-66 T3-89 T4-22	NA	NA	NA	NA	HG-274 LG-123	Y-94	NA	2.2 (0.6-5.0)	-T stage -Surgical approach -LN stage -Grade -Surgical margin	OS DSS RFS	6
44.	Fang 2018 China	612	R	N	Pelvis-65.29 +/− 11.11 Ureter-68.07 +/− 10.20	340/272	NA	41	pTa-1=206 pT2-4=406	NA	75	NA	P-341 U-271	G1-19 G2-334 G3-259	NA	NA	64	-Necrosis -LN status -Architecture -Grade -CIS	OS CSS	7
45.	Gao 2017 China	259	R	N	67.53	187/179	O-80 L-179	24	<=pT2-171>=pT3-88	212	NA	NA	NA	G1-59 G2-3=200	NA	23 (8.8%)	33.3 (15.5-64.2)	-AST/ALT -Stage -Grade -Histology -Sarcomatoid differentiation	OS PFS CSS Bladder recurrence free survival	7
46.	Godfrey 2012 USA	211	R	N	70 (11.4)	124/87	O-121 L-90	59	Ta-Tis=78 T1-41 T2-18 T3-71 T4-3	68	NA	NA	P-170 U-41	HG-134 LG-77	NA	NA	27 (11-65.5)	-Race -LVI -High nuclear grade	OS OSS	6
47.	Hara 2015 Japan	1172	R	Y	NA	806/366	O-750 L-421 Missing data-1	1138	Ta-125 Tis-29 T1-344 T2-302 T3-240 T4-21 Tx-111	423	NA	NA	P-593 U-546 Both-32 Missing data-1	G0-1 G1-71 G2-528 G3-558 Missing data-14	179	NA	55.8	-Age -Stage -LN -Metastasis -LVI -Infiltrative growth pattern	OS RFS	7
48.	Inamoto 2011 Japan	103	R	N	68.6 ±10.05	71/32	NA	Y	Tis/Ta/T1-43 T2-13 T3/T4-47	32	Nil	NA	-	G1-20 G2-28 G3-55	-	11	29 (14-63)	-C reactive protein -BMI -Focality -Lymph.Node	OS CSS RFS	6
49.	Saito 2007 Japan	189	R	N	NA	94/41	NA	Y	≤T2-73 T3-62	57	Nil	NA	59/76	LG-81 HG-54	30	-	55 (3-232)	-Age-pT-LVI	CSS RFS	6
50.	Sakano 2014 Japan	502	R	Y	72 (32-93)	344/158	NA	Y	<3-290 ≥3-212	166	Nil	NA	221/232	LG-257 HG-233	144	60	41.4 (3-200)	-pT -Grade -LVI -Variant Histology	CSS	7
51.	Shibing 2015 China	417	R	N	67 (26-86)	246/171	NA	Y	Tis/Ta/T1-118 T2-79 T3-168 T4-52	74	Nil	NA	271/110	LG-100 HG-317	78	90	26 (12-54)	-pT -Grade -L.Nodes -Tumor Size -SurgicalMargins	OS CSS RFS	7
52.	Song 2019 Korea	453	R	N	69 (52-80)	320/133	O-164 L-143 Robotic-146	Y	Ta-6 T1-127 T2-147 T3-145 T4-23	132	Nil	NA	161/201	G1-2 G2-225 G3-222	-	-	23.2 (0-172)	-BMI -pT -LVI -L.Node -HDN -HTN	OS CSS RFS	7
53.	Sung 2014 Korea	386	R	N	64 (56-71)	293/93	NA	Y	Ta/Tis-78 T1-85 T2-56 T3/T4-167	-	Nil	NA	175/166	G1-20 G2-193 G3-161	-	7	39 (21.1-70.6)	-Age -Gender -Location -Grade -pT	RFS CSS	7
54.	Tai 2015 Taiwan	503	R	N	68 (60-74.8)	249/254	NA	Y	Ta/Tis/T1-144 T2-31 T3-101 T4-4	49	Nil	NA	280/184	LG-135 HG-142	-	-	52 (23-77)	-Grade -pT -LVI -Location	OS RFS CSS	6
55.	Tan 2018 China	668	R	Y	65.8 (54.4-77.2)	380/288	NA	Y	≤ pT2-338 ≥ pT3-330	99	Nil	NA	353/196	LG-173 HG-495	281	-	45 (21-74)	-Focality -pT -L.Nodes -LVI -LDH	CSS OS RFS MFS	7
56.	Tanaka 2012 Japan	218	R	Y	69 (38-92)	160/5 8	O-155 L-63	Y	Ta-T1-75 T2-27 T3-107 T4-9	84	Nil	NA	130/88	LG-59 HG-159	42	-	38 (3-187)	-Plasma Fibrinogen -pT -LVI	CSS RFS	7
57.	Tanaka 2015 Japan	394	R	Y	70 (63-77)	289/105	NA	Y	Ta/T1-125 T2-57 T3-201 T4-11	170	Nil	NA	232/162	LG-128 HG-266	88	-	30 (15-63)	-pT -LVI -Plasma Fibrinogen	CSS RFS ACM	7
58.	Tang 2015 China	687	R	N	68 (20-90)	306/381	NA	Y	T1-216 T2-217 T3-160 T4-13	-	Nil	NA	339/267	G1-20 G2-354 G3-232	-	81	65 (3-144)	-Gender -pT -Variant Histology -Pre op -HDN	RFS CSS	7
59.	Vartolomei 2015 Multicentre	2274	R	Y	69 (61-76)	1527/747	NA	Y	Ta-497 Tis-48 T1-532 T2-441 T3-671 T4-85	499	516	-	1448/826	LG-367 HG-1907	-	-	40 (20-76)	-pT -Grade -LVI -NLR -L.Node -Gender	RFS CSS	7
60.	Waseda 2015 Japan	1068	R	Y	70 (62-76)	758/310	NA	Y	Ta-127 Tis-34 T1-186 T2-164 T3-518 T4-39	446	Nil	NA	198/181	LG-751 HG-317	-	-	40 (17-77)	-Age -LVI -pT -pN -Location	RFS CSS	6
61.	Xu 2018 China	662	R	N	67 (59-74)	376/286	O-430 L-232	Y	≤pT2-338 >pT3-324	100	Nil	NA	349/193	LG-169 HG-493	279	149	42 (19-72)	-Grade -pT -L.Node -Variant Histology -CONUT score	OS RFS CSS	6
62.	Shibing 2016 China	795	R	Y	NA	462/333	O-588 L-207	Y	Tis/Ta/T1-149 T2-241 T3-313 T4-92	169	Nil	NA	497/187	LG-212 HG-583	202	162	32 (17-60)	-Grade -pT -LVI -Variant Histology -Size -Lymph.Node	OS CSS RFS	7
63.	Zamboni 2019 Multicentre	1610	R	Y	69 (61-76)	1096/512	O-999 L-489	Y	T0/Ta/Tis-401 T1-330 T2—227 T3-521 T4-110	344	235	NA	NA	HG-1058	233	150	42	-micropapillary variant -T3-4 stage -Sarcomatoid variant	RFS CSM	6
		35714																		

R-Retrospective, U- ureter, P-Renal Pelvis, O- Open, L- Laparoscopic, R- retrospective , LG- low grade, HG- high Grade, G-grade , LVI-Lymphovascular invasion, STSM- soft tissue surgical margin, T stage- pathological T stage, INF- interferon, O-Open, L= Laparoscopic, X= not known, LN- Lymph node, AST- aspartate transaminase, ALT-alanine transminase, CSS- cancer specific survival, RFS- Recurrence free survival, OS- overall survival, MFS-metastasis free survival, ECOG- Eastern co-operative oncology group, HB- hemoglobin, GFR- Glomerular filtration rate, CIS- carcinoma in situ.
